# Seasonal lipid dynamics of four Arctic bivalves: Implications for their physiological capacities to cope with future changes in coastal ecosystems

**DOI:** 10.1002/ece3.10691

**Published:** 2023-11-02

**Authors:** Guillaume Bridier, Frédéric Olivier, Jacques Grall, Laurent Chauvaud, Mikael K. Sejr, Réjean Tremblay

**Affiliations:** ^1^ Institut des Sciences de la mer de Rimouski Université du Québec à Rimouski Rimouski Quebec Canada; ^2^ Biologie des Organismes et Ecosystèmes Aquatiques (BOREA) UMR 8067 MNHN, CNRS, SU, IRD 207, UCN, UA Paris France; ^3^ Laboratoire des Sciences de l'Environnement Marin (LEMAR) UMR 6539 UBO, CNRS, IRD, Ifremer Plouzané France; ^4^ Observatoire Marin de l'Institut Universitaire Européen de la Mer UMS 3113, Université de Bretagne Occidentale Plouzané France; ^5^ Arctic Research Centre and Ecoscience Aarhus University Aarhus C Denmark

**Keywords:** Arctic coastal ecosystems, bivalve, climate change, energy reserves, fatty acids, homeoviscous adaptation, meltwater inputs, metabolic rate depression

## Abstract

The Arctic is exposed to unprecedented warming, at least three times higher than the global average, which induces significant melting of the cryosphere. Freshwater inputs from melting glaciers will subsequently affect coastal primary production and organic matter quality. However, due to a lack of basic knowledge on the physiology of Arctic organisms, it remains difficult to understand how these future trophic changes will threaten the long‐term survival of benthic species in coastal habitats. This study aimed to gain new insights into the seasonal lipid dynamics of four dominant benthic bivalves (*Astarte moerchi*, *Hiatella arctica*, *Musculus discors*, and *Mya truncata*) collected before and after sea ice break‐up in a high‐Arctic fjord (Young Sound, NE Greenland). Total lipid content and fatty acid composition of digestive gland neutral lipids were analyzed to assess bivalve energy reserves while the fatty acid composition of gill polar lipids was determined as a biochemical indicator of interspecies variations in metabolic activity and temperature acclimation. Results showed a decrease in lipid reserves between May and August, suggesting that bivalves have only limited access to fresh organic matter until sea ice break‐up. The lack of seasonal variation in the fatty acid composition of neutral lipids, especially essential ω3 fatty acids, indicates that no fatty acid transfer from the digestive glands to the gonads occurs between May and August, and therefore, no reproductive investment takes place during this period. Large interspecies differences in gill fatty acid composition were observed, which appear to be related to differences in species life span and metabolic strategies. Such differences in gill fatty acid composition of polar lipids, which generally influence metabolic rates and energy needs, may imply that not all benthic species will be equally sensitive to future changes in primary production and organic matter quality in Arctic coastal habitats.

## Graphical abstract

This study aimed to gain new knowledge on the physiology of four Arctic benthic bivalves exposed to high seasonal trophic constraints by using fatty acids as indicators of nutritional status, energy requirements, and metabolic rates of bivalves. Results highlight the importance of life traits such as longevity and metabolism for the capability of benthic invertebrates to cope with poor trophic conditions (i.e., in terms of resource quality/quantity). Results suggest that long‐living and low‐metabolism species may be more resilient to future changes in trophic environment quality in Arctic ecosystems due to their lower energetic turnover and reduced essential fatty acid requirements.

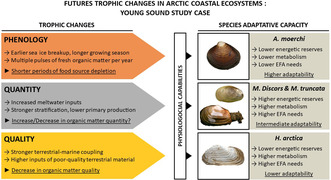

## 1 INTRODUCTION

Arctic ecosystems are undergoing unprecedented changes due to atmospheric warming at a rate of three to four times higher than the global average (Rantanen et al., 2022). Since the 1970s, rising temperatures have led to a 40% reduction in summer (September) sea ice extent and a 65% reduction in sea ice thickness (AMAP, 2017; Kwok, 2018). These environmental changes have caused a shift in phytoplankton phenology (Ardyna et al., 2014) and a 57% increase in phytoplankton productivity between 1998 and 2018 (Lewis et al., 2020). However, these trends mask strong disparities at smaller spatial scales during the same period, as primary production has increased very strongly on inflow shelves (e.g., Fram Strait, Bering Sea), while remaining stable or decreasing on outflow shelves (e.g., Canadian Arctic Archipelago, Ardyna & Arrigo, 2020; Tremblay et al., 2015). The effects of climate change are also heterogeneous in coastal areas, which are exposed to both specific local (e.g., freshwater inputs) and broader constraints acting at the entire Arctic scale (Bridier, 2020; Wassmann et al., 2020). Inputs of meltwater and terrestrial particles in coastal areas modify the composition of phytoplankton and bacterial communities through changes in seawater stratification and turbidity (Arendt et al., 2010; Delpech et al., 2021; Paulsen et al., 2017), which reduces phytoplankton production (Holding et al., 2019; Meire et al., 2017; Murray et al., 2015). In addition, meltwater inputs provide refractory terrestrial organic carbon from melting glaciers and thawing permafrost to coastal marine ecosystems (Hernes et al., 2021; Wadham et al., 2019). All these environmental changes subsequently affect the coastal food webs by changing the origin of food sources and reducing their quality, in terms of polyunsaturated fatty acids (PUFAs), available to benthic primary consumers (Bridier et al., 2019; Kędra et al., 2015). Such a decrease in PUFA from organic matter could have subsequent consequences on the physiology of benthic invertebrates, especially since some PUFAs are considered essential (essential fatty acids (EFAs), e.g., 20:5ω3, 22:6ω3). EFAs are recognized to be weakly synthesized de novo by marine invertebrates but are essential for their growth and survival through their crucial role in physiological function (Parrish, 2009).

The fatty acids stored in reserve tissues (the neutral lipid fraction) serve as the main energy reserve in benthic invertebrates and are mobilized to meet metabolic needs (Haider et al., 2020; Mayrand et al., 2017; Pazos et al., 1997), or to support growth and reproduction (e.g., egg production, Leroy et al., 2013). In contrast to fatty acids used as energy stores, those incorporated into cell membranes (the polar lipid fraction) play a crucial role in the functioning of the organism by maintaining the integrity of cell membranes, particularly in relation to changes in temperature. In poikilotherm animals, the fluidity of cell membranes decreases at lower temperatures (Hochachka & Somero, 2002). Reduced membrane fluidity leads to a decrease in the activity of membrane‐bound proteins and ion diffusion rates, ultimately resulting in a decrease in metabolism (Hochachka & Somero, 2002). To counteract these effects, poikilotherms can adjust their membrane lipid composition when they are exposed to cold temperature (a process called *Homeoviscous adaptation*) by increasing the proportion of monounsaturated fatty acids (MUFAs) and PUFAs relative to saturated fatty acids, lengthening the carbon chains of fatty acids, or decreasing the cholesterol content of cell membranes (Hazel, 1995; Pernet et al., 2006; Sinensky, 1974). According to the *membrane‐pacemaker theory of aging* (Hulbert, 2005; Hulbert et al., 2007), the fluidity of cell membranes is also influenced by their fatty acid composition, which affects the metabolic level and longevity of the organism. A high metabolic level promotes the formation of reactive oxygen species that are prone to damage cell membranes, especially phospholipids composed of PUFA (Kraffe et al., 2004). To avoid the oxidation of lipid membranes, long‐lived species reduce the level of unsaturation in their phospholipids (i.e., the number of double bonds of their fatty acids) with subsequent negative consequences on the metabolic rate (Abele et al., 2009; Blier et al., 2017; Hulbert et al., 2007).

In this context, the presence of sufficient amounts of EFA in organic matter sources ingested by primary consumers is crucial to maintain the integrity of their cell membranes and to ensure their optimal physiological function. Thus, the availability of EFA in organic matter might be expected to ultimately determine the survival of some species in their environment. However, because of the relatively limited understanding of the physiology of Arctic species and their requirements for EFA, it remains a challenge to determine their vulnerability to future trophic changes in coastal ecosystems. This lack of fundamental knowledge is particularly evident for benthic invertebrates (Renaud et al., 2015, 2019), which are less studied than other taxa such as fish or seabirds (Poloczanska et al., 2016), despite their key role in Arctic marine food webs (e.g., Grebmeier, 2012; Kędra et al., 2015). Therefore, the objectives of this study are to: (1) provide new basic information on the physiology of bivalves with different longevity (e.g., cold acclimation and metabolism), (2) identify some basis of physiological requirements of benthic invertebrates in terms of EFAs, and (3) propose new hypotheses on the physiological capacity of these species to cope with future changes in trophic environment quality in Arctic ecosystems.

## 2 MATERIALS AND METHODS

### 2.1 Study site and choice of model species

**FIGURE 1:**
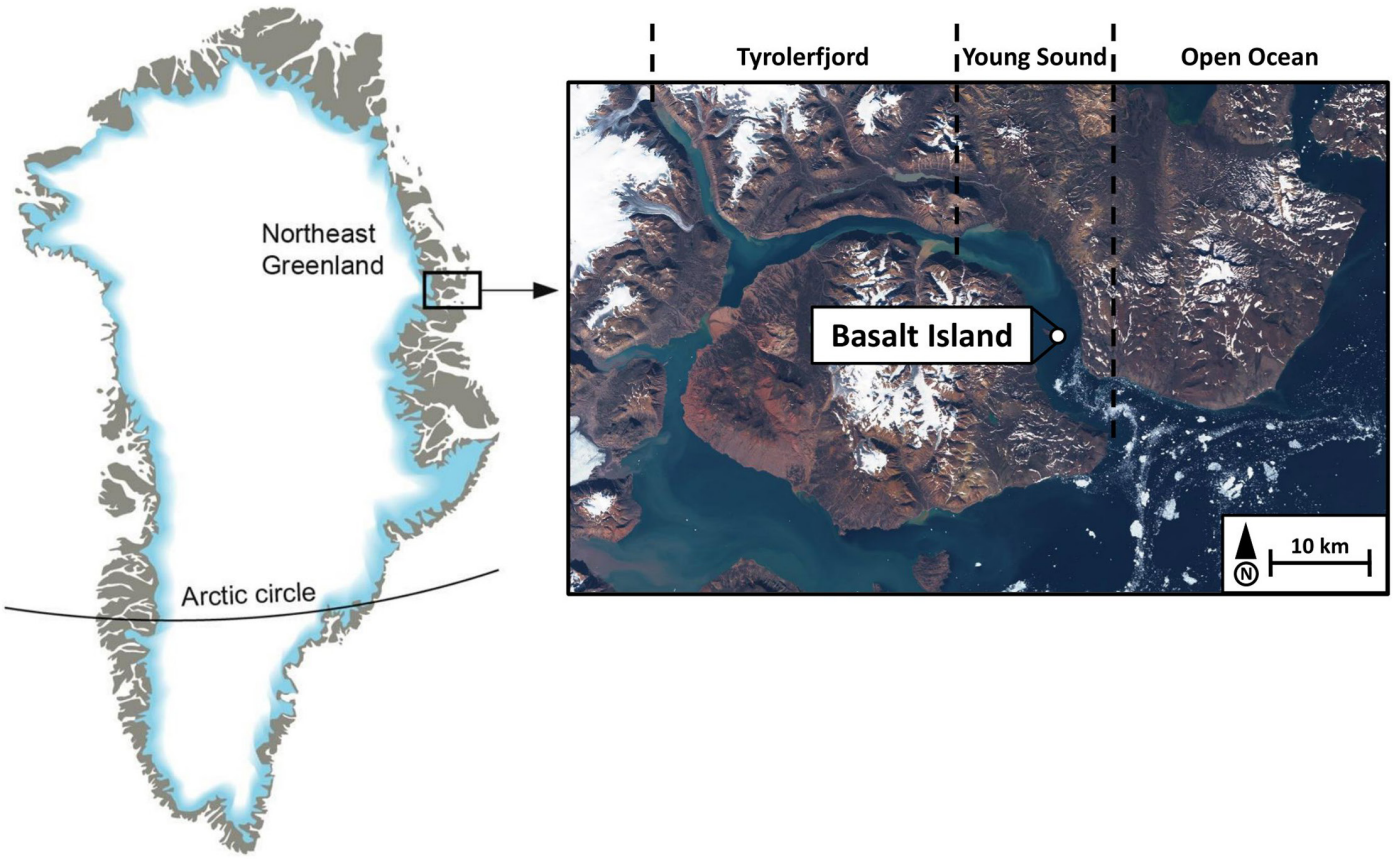
Map of the study site showing the location of the Young Sound‐Tyrolerfjord system and the Basalt Island sampling station.

Samples were collected in Young Sound, a High‐Arctic fjord that is part of the Young Sound‐Tyrolerfjord system located in the northeast Greenland (74.2–74.3° N, 19.7–21.9° W, Figure 1). Due to the long duration of sea ice cover (9–10 months per year), this fjord is considered one of the least productive coastal ecosystems in the world, with pelagic primary production estimated at only 10 g C m^−2^ year^−1^ (Rysgaard et al., 1999). The Young Sound‐Tyrolerfjord system is 90 km long, varies in width between 2 and 7 km, and covers an area of 390 km^2^ (Rysgaard et al., 2003). The maximum depth is 330 m while the average depth is around 100 m (Rysgaard et al., 2003). The Young Sound‐Tyrolerfjord system is characterized by two shallow sills, the outermost of which is 45 m deep and separates the water masses of the fjord from those of the Greenland Sea (Bendtsen et al., 2007). The fjord is exposed to meltwater inputs from June to September, to total volume of which is estimated to be about 0.9–1.4 km^3^ per year (Bendtsen et al., 2014). The land‐terminating glaciers contribute significantly to this discharge, accounting on average for 50%–80% of the annual freshwater inputs, especially in the inner region of the fjord (Citterio et al., 2017). These summer meltwater plumes induce strong spatial gradients in salinity and temperature in surface waters above the pycnocline (i.e., 5–10 m), ranging from 8 to 30 PSU and from to 7 to 2°C, respectively, between the inner and outer fjords (Bendtsen et al., 2014; Rysgaard et al., 2003; Sejr et al., 2017). However, subsurface and bottom waters (i.e., below the pycnocline) remain stable throughout the year (temperature < to 0.5°C and salinity >27 PSU throughout the year at 17 m, Sejr et al., 2022).

Such thermal stability of bottom water masses thus provides an opportunity to study the impact of seasonality on bivalve physiology from a trophic (organic matter quality and availability) and reproductive perspective. Four Arctic bivalve species characterized by different longevity were selected: *Astarte moerchi* (maximum longevity [ML] ≈ 110 years, Olivier et al., 2020), *Hiatella arctica* (ML ≈ 120 years, Sejr et al., 2002), *Musculus discors* (ML ≈ 10 years, Selin, 2010), and *Mya truncata* (ML ≈ 50 years, Sejr & Christensen, 2007). These four bivalve species are all suspension feeders and are among the most abundant mollusks in Young Sound, with densities reaching up to 47 inds.m^−2^ for *A*. *moerchi*, 803 inds.m^−2^ for *H*. *arctica*, 1882 inds.m^−2^ for *M*. *discors*, and 66 inds.m^−2^ for *M*. *truncata* (Bridier, 2020; Sejr et al., 2000).

Little information is available on the reproduction of Arctic bivalve in general, except that spawning periods are usually strongly synchronized with the onset of phytoplankton blooms (Brandner et al., 2017). However, these species can be divided into two distinct groups according to the mode of larval development. Larvae produced by *H*. *arctica* and *M*. *truncata* have a pelagic development (with wide larval dispersal), whereas those produced by *A*. *moerchi* and *M*. *discors* have a benthic development (i.e., without larval dispersal, Brandner et al., 2017; Larsen et al., 2007). A specific study carried out on *H*. *arctica* shows that this species spawns between June and October in Young Sound (Veillard et al., 2023). The reproductive cycles of *M*. *truncata* and *H*. *arctica* are generally synchronized in the Arctic (Brandner et al., 2017), which could suggest that these two species also spawn at the same time in Young Sound (i.e., from June to the end of October). However, to our knowledge, there is no information on the reproductive strategies of *M*. *discors* and *A*. *moerchi* in the literature.

### 2.2 Sampling

Bivalves were collected at the Basalt Island station (depth = 21.5 m, 74.33° N, 20.36° W, see Figure 1) in May 2018 under approximately 2 m of sea ice cover (Laurent Chauvaud, personal observations) by SCUBA divers and in August 2018 (only 2–3 days after sea ice breakup) using a 0.04 m^2^ Van Veen grab. Although winter and summer bivalves were collected using two different methods (divers vs. Van Veen grab), both were conducted randomly (i.e., without selection of a specific area or a bivalve size). As the aim of this study was not to compare species abundances or densities (which would obviously be influenced by the sampling methods), but only to gain new insights into the physiology of Arctic bivalves, we believe that these two sampling methods will not influence our seasonal comparisons of bivalve lipid profiles. Unlike other species, the grab does not allow sufficient penetration into the sediment to collect *M*. *truncata*. Instead, *M. truncata* specimens were collected by scuba divers during a field campaign conducted in August 2015 (to avoid methodological bias, seasonal variations are not discussed for this species). After collection, samples were stored in the field (Daneborg Station) in a −80°C freezer, then transferred to Canada in a −20°C cooler case and stored in a −80°C freezer at UQAR/ISMER until analysis.

### 2.3 Lipid analyses

A total of 76 individuals (23 *A*. *moerchi*, 20 *H*. *arctica*, 18 *M*. *discors*, and 15 *M*. *truncata*) were dissected in the laboratory to collect digestive glands and gills and estimate their wet weight prior to lipid extraction. Digestive gland weights and sizes of dissected individuals are provided in Table S1. Individuals were not sexed prior to lipid analyses. Although sex can control the lipid composition of specific tissues (e.g., gonads, mantle), it generally does not influence the lipid profiles of gills and digestive glands (e.g., Birkely et al., 2003; Fernández‐Reiriz et al., 2015). To limit potential life stage bias affecting the interindividual variability in lipid profiles, we decided to analyze only individuals that had reached adult size (i.e., assumed to be sexually mature). In addition, all individuals collected for each species were generally evenly distributed across several size classes to avoid an effect of size and/or age on the interindividual variability in lipid profiles. This effect is moreover known to be negligible compared to interspecies physiological differences in mollusk bivalves (Munro & Blier, 2015).

In the digestive gland, the neutral lipid fraction was used as a proxy for food availability and quality because dietary fatty acids are incorporated into triacylglycerol storage lipids with almost no modification (Couturier et al., 2020; Dalsgaard et al., 2003). For gills, only the polar lipid fraction was recovered and used as a proxy for metabolic rate and acclimation to cold temperature since gill polar lipids are mostly composed of fatty acids from phospholipids involved in the good physiological functioning of organisms (Couturier et al., 2020; Hazel & Williams, 1990; Pernet et al., 2007). Lipid extractions were performed according to the method of Folch et al. (1957) as modified in Chen et al. (1981). Lipids were extracted by grinding digestive glands and gills in tissue grinders with a dichloromethane/methanol solution (2:1, v:v). Total lipid extracts from digestive glands were weighed prior to separation of lipid fractions for use as a proxy for the nutritional status of the bivalves. Neutral and polar fractions were separated on silica gel columns hydrated with 6% deionized water. The columns were first preconditioned with 10 mL of methanol and 10 mL of dichloromethane. Dry lipid extracts were retrieved three times in 0.5 mL of dichloromethane (total volume = 1.5 mL) to ensure that no lipids remained in the tubes and then loaded onto the upper part of silica columns. Silica columns containing lipid extracts (i.e., neutral and polar fractions) were first washed with 10 mL dichloromethane/methanol (98:2, v:v) to elute neutral lipids and then washed with 10 mL methanol to elute polar lipids (Marty et al., 1992). Eluted fractions were evaporated under a N2 flow, diluted in a methylation solution (Butyl‐hydroxytoluene/sulfuric acid/toluene), heated at 100°C for 10 min to methylate fatty acids and purified on silica gel columns to remove free sterols (Mejri et al., 2014). Fatty acids were finally separated on an Omegawax 250 capillary column (Supelco) and determined by gas chromatography (Trace GC Ultra, Thermo Scientific) coupled to a mass detector (model ITQ900, Thermo Scientific). Fatty acids were identified using commercial standards (Supelco® 37 component FAME mix, CRM47885, Sigma‐Aldrich) or mass spectra for peaks not assigned to a known standard using Xcalibur v 3.1 software (Thermo Scientific).

Two indicators based on the fatty acid composition of the gills were calculated to assess the thermal acclimation of bivalves to cold temperatures and their physiological requirements of EFAs. First, thermal acclimation was assessed by the unsaturation index (UI) based on the equation proposed by Logue et al. (2000):

$$\begin{array}{c} \mathrm{UI}=\left(1\times \%\text{Monoenoics}\right)+\left(2\times \%\text{Dienoics}\right)+\left(3\times \%\text{Trienoics}\right)\\ +\left(4\times \%\text{Tetraenoics}\right)+\left(5\times \%\text{Pentaenoics}\right)+\left(6\times \%\text{Hexaenoics}\right) \end{array}$$

UI represents the average number of double bonds (i.e., unsaturation) between the carbons of a single fatty acid multiplied by 100 (Hazel & Williams, 1990). An UI of 100 would mean that each fatty acid has on average a single unsaturation, while an UI of 300 would mean that each fatty acid has three double bonds. The UI does not provide a direct measure of membrane fluidity (i.e., permeability of membrane proteins), but it is a useful proxy for comparing membrane fluidity between different species and relating these biochemical differences to differences in metabolism or cold acclimation (Hulbert et al., 2007; Munro & Blier, 2015; Pernet et al., 2007). A species with a higher UI (i.e., higher membrane fluidity) is expected to have a higher metabolism and to be more acclimated to low temperatures. Conversely, a species with a low UI (i.e., low membrane fluidity) is considered to have a lower metabolism and to be less acclimated to low temperatures (Munro & Blier, 2015, Pernet et al., 2007). Second, the potential physiological requirements of bivalves for EFAs were estimated by calculating an index based on the ratio of fatty acids present in gill polar lipids (structural lipids) to the fatty acids from total lipids of food sources (usually particulate organic matter [POM]), as described by the following formula (example with fatty acid 20:ω3):

$$\text{Gill}:\text{Diet ratio}=\frac{\%\text{of}\;20:\omega 3\;\text{in}\;\mathrm{POM}\;\text{total lipids}}{\%\text{of}\;20:5\omega 3\;\text{in gill polar fatty acids}\;}$$

This comparison allows us to assess whether specific fatty acids are selectively retained by the organism to ensure the integrity of cell membranes (Copeman et al., 2002; Gendron et al., 2013; Mejri et al., 2021). If the relative proportion of a specific fatty acid in gill polar lipids is equal to or greater than the dietary level (i.e., Gill:Diet ratio equal to or less than 1), then the bivalve requirement for that specific fatty acid is presumably met. Conversely, if the Gill:Diet ratio of a fatty acid is greater than 1, it means that this specific fatty acid is selectively incorporated by bivalve and thus reveals a potential dietary deficiency. To calculate this ratio, we used data from Bridier et al. (2019) on the fatty acid composition of particulate organic matter filtered by bivalves in Young Sound as a proxy for the lipid profile of food sources consumed by the bivalves sampled in this study. We chose to use only summer fatty acid profiles of gill polar lipids and total lipids of particulate organic matter to calculate Gill:Diet ratios, as it has already been shown in Young Sound that bivalves feed only marginally in winter due to depletion of the trophic environment (Bridier et al., 2019).

### 2.4 Statistical analyses

Data for total lipid content of digestive glands were analyzed using a univariate two‐way PERMANOVA (species and season as factors). The PERMANOVAs were preferred to classical ANOVA tests because they do not require assumptions of normality and homoscedasticity for balanced designs (Anderson & Walsh, 2013). When significant, we used a pairwise PERMANOVA to determine whether the observed differences were significant across all groups (i.e., all species and/or seasons). Seasonal and interspecies variation in the fatty acid composition of both neutral and polar lipids was visualized using two principal component analyses (PCAs). The PCAs were based on transformed fatty acid data using the Hellinger distance to reduce the weight of rare fatty acids on the ordination (Legendre & Gallagher, 2001). The effects of season, species, and their interactions on the fatty acid composition of neutral and polar lipids were assessed by separate multivariate two‐way PERMANOVAs. In addition, univariate two‐way PERMANOVAs and pairwise PERMANOVAs were applied for lipid content in digestive glands, lipid indicators (UI; sum of saturated fatty acids (SFAs), MUFA (monounsaturated fatty acids), PUFA, branched fatty acids (BrFAs), EFA, 20:2 & 22:2 NMI (non‐methylene‐interrupted fatty acids) and unknown PUFA; MUFA/PUFA, and EPA/DHA (i.e., 20:5ω3/22:6ω3) ratios) as well as each fatty acid to test their specific variability between the two seasons and four species. Univariate one‐way PERMANOVAs with species as factor were applied for Gill:Diet ratios of specific fatty acids (i.e., 20:5ω3 and 22:6ω3) and lipid indicators (i.e., sum of SFA, MUFA, and PUFA), since Gill:Diet ratios were calculated only for the summer season. All statistical analyses were performed using R (R Core Team, 2019) and the “vegan” package (Oksanen et al., 2019).

## 3 RESULTS

### 3.1 Nutritional level status indicator

**FIGURE 2:**
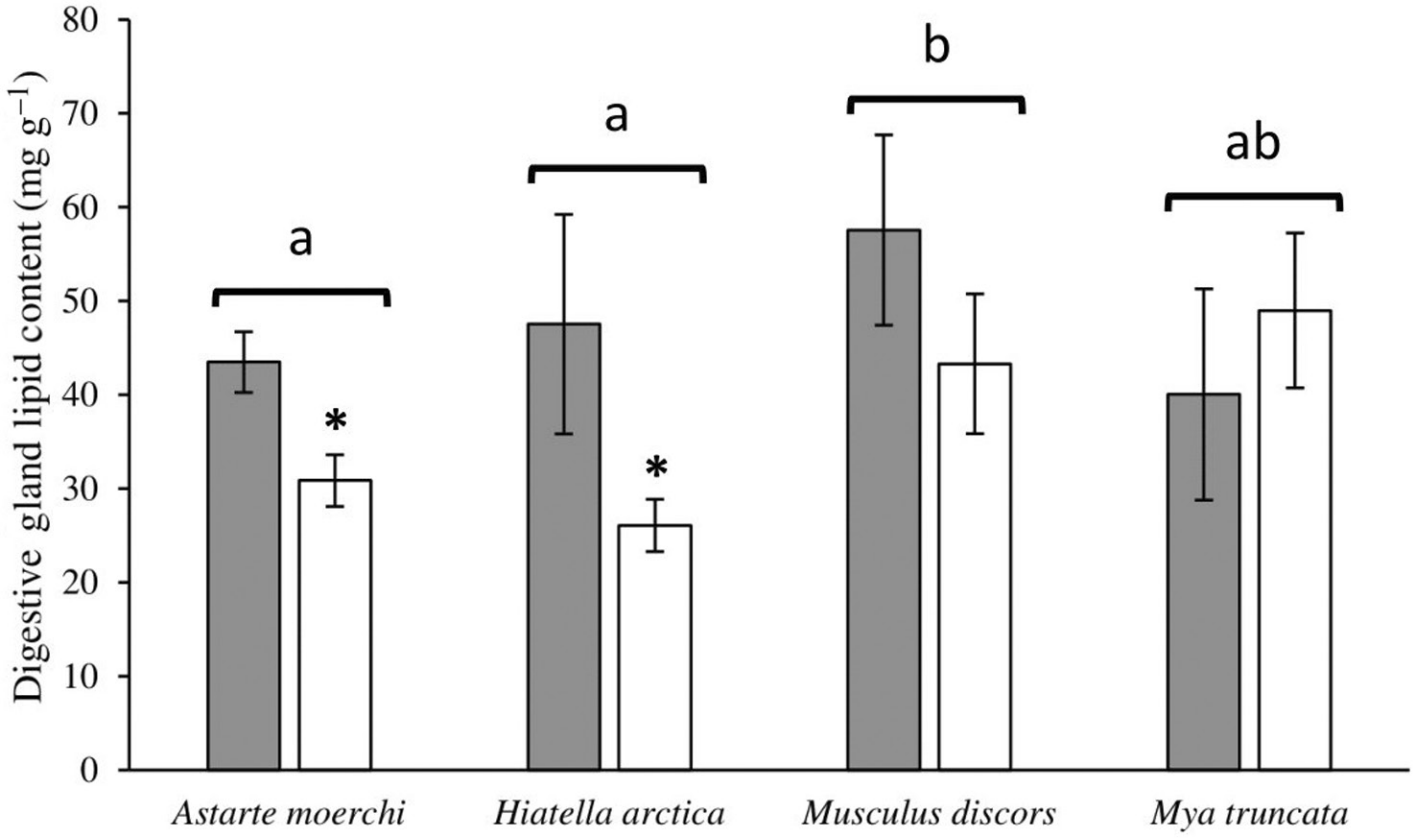
Seasonal variation in digestive gland lipid contents (mg per g of wet weight) of four bivalve species. Data are mean ± Standard error of the mean (SEM). Letters indicate significant interspecific variation in lipid content (winter and summer seasons combined), asterisks indicate significant seasonal variation between lipid content of a same species, and N is the number of replicates per species and season.

The winter lipid contents in the digestive glands were 40.0 ± 11.3 mg g^−1^ for *Mya truncata* (mean ± standard error of the mean), 43.5 ± 3.2 mg g^−1^ for *Astarte moerchi*, 47.5 ± 11.7 mg g^−1^ for *Hiatella arctica*, and 57.6 ± 10.1 mg g^−1^ for *Musculus discors* (Figure 2). The summer lipid contents in the digestive glands were 26.1 ± 2.8 mg g^−1^ for *H*. *arctica*, 30.9 ± 2.8 mg g^−1^ for *A*. *moerchi*, 43.3 ± 7.7 mg g^−1^ for *M*. *discors*, and 49.0 ± 8.3 mg g^−1^ for *M*. *truncata* (Figure 2).

The total lipid content of the digestive glands showed variability between the two seasons (degrees of freedom for treatment and error [DF] = 1 and 47, pseudo‐*F* = 1931.92, *p*‐perm = .007) and between the four species (DF = 3 and 47, pseudo‐*F* = 902.68, *p*‐perm = .022) without interactions between these two factors (DF = 3 and 47, pseudo‐*F* = 384.61, *p*‐perm = .216). The levels were significantly higher in the digestive glands of *M*. *discors* than in the glands of *A*. *moerchi* and *H*. *arctica* (*p* < .05) while *M*. *truncata* showed intermediate values (Figure 2, Table S1). A seasonal effect was observed in the digestive gland from *A. moerchi* and *H. arctica* decreasing by more than 35% between winter and summer (*p* < .05) while no seasonal effect was observed for *M*. *discors* and *M*. *truncata* (Figure 2).

### 3.2 Neutral lipid profiles in digestive glands

Overall, neutral lipid profiles showed high levels of PUFAs, ranging from 40.5 ± 3.0% (*A*. *moerchi*) to 46.6 ± 0.9% (*M*. *discors*) in winter, and from 43.0 ± 3.0% (*H*. *arctica*) to 47.6 ± 1.3% (*M*. *truncata*) in summer (Table S2). These high levels of PUFAs are mainly explained by the high levels of 20:5ω3 and 22:6ω3. Levels of 20:5ω3 varied from 2.7 ± 0.5% (*M*. *truncata*) to 4.8 ± 0.3% (*H*. *arctica*) in winter and from 3.4 ± 0.3% (*M*. *truncata* and *M*. *discors*) to 5.6 ± 0.5% (*H*. *arctica*) in summer (Table S2). Levels of 22:6ω3 varied from 24.6 ± 3.0% (*A*. *moerchi*) to 33.8 ± 2.7% (*M*. *truncata*) in winter and from 24.4 ± 1.1% (*H*. *arctica*) to 37.0 ± 0.5% (*M*. *truncata*) in summer (Table S2).

**FIGURE 3:**
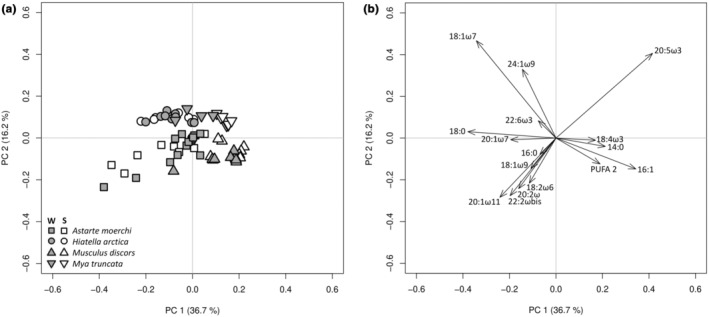
Principal component analysis (PCA) based on Hellinger‐transformed neutral fatty acid percentages of bivalve digestive glands from winter (W) and summer (S) seasons (a: individual factor map, b: variable factor map). Variable factor map includes only the 15 most discriminant fatty acids.

The two‐way PERMANOVA showed no variation between the two sampled seasons (DF = 1 and 62, pseudo‐*F* = 1.3410, *p*‐perm = .2367) and no interactions between species and season factors (DF = 3 and 62, pseudo‐*F* = 1.6413, *p*‐perm = .1184). However, significant differences were observed between the four species (DF = 3 and 62, pseudo‐*F* = 12.1583, *p*‐perm = .0001). According to pairwise comparisons, lipid profiles in the neutral fraction of digestive glands were significantly different between all species (*p* < .05). The PCA showed that 36.7% of the total fatty acid variation was explained by the first principal component (Figure 3a,b). The first principal component discriminated the lipid profiles of *A. morchi* and *H. arctica* from those of *M. discors* and *M. truncata* while the second principal component (explaining 16.2% of the total variation) distinguished *A. moerchi* and *M. discors* from *H. artica* and *M. truncata* (Figure 3a). Overall, the fatty acid composition of the neutral fraction of digestive glands from all species was first dominated by a high relative contribution of 20:5ω3 (ranging from 24.4 ± 1.1% [*H*. *arctica*] to 37.0 ± 0.5% [*M*. *truncata*]), followed by 16:1ω7 (ranging from 13.7 ± 1.0% [*H*. *arctica*] to 24.6 ± 1.0% [*M. discors*]), then 16:0 (ranging from 10.0 ± 1.6% [*M*. *truncata*] to 14.1 ± 0.6% [*H*. *arctica*]) (Table S2).

### 3.3 Polar lipid profiles in gills

Overall, the polar lipid profiles showed high levels of PUFAs, ranging from 40.1 ± 3.2% (*A*. *moerchi*) to 61 ± 1.6% (*H*. *arctica*) in winter and from 39.0 ± 3.0% (*A*. *moerchi*) to 63.7 ± 1.1% (*M*. *truncata*) in summer (Table S3). These high levels of PUFAs are mainly explained by the high levels of 20:2 NMI, 20:5ω3, and 22:6ω3. Levels of 20:2 NMI ranged from 8.0 ± 0.6% (*A*. *moerchi*) to 12.0 ± 0.8% (*H*. *arctica*) in winter and from 7.8 ± 0.7% (*A*. *moerchi*) to 13.0 ± 0.9% (*M*. *truncata*) in summer (Table S3). Levels of 20:5ω3 varied from 3.7 ± 1.2% (*A*. *moerchi*) to 14.9 ± 1.9% (*M*. *truncata*) in winter and from 3.9 ± 0.8% (*A*. *moerchi*) to 11.2 ± 0.7% (*M*. *discors*) in summer (Table S3). Finally, levels of 22:6ω3 varied from 3.6 ± 1.2% (*A*. *moerchi*) to 15.2 ± 0.8% (*H*. *arctica*) in winter and from 3.0 ± 0.8% (*A*. *moerchi*) to 16.2 ± 0.5% (*M*. *truncata*) in summer (Table S3).

**FIGURE 4:**
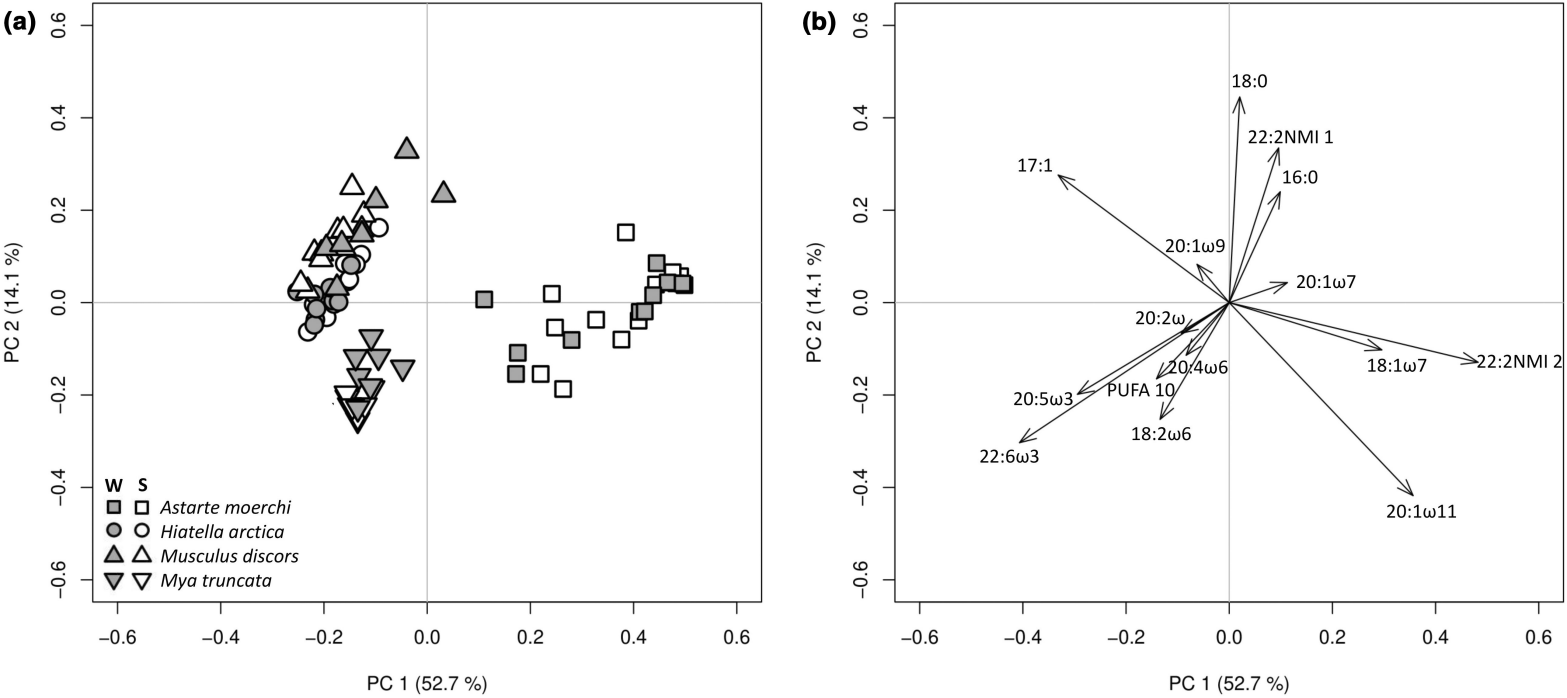
Principal component analysis (PCA) based on Hellinger‐transformed polar fatty acid percentages of bivalve gills from winter (W) and summer (S) seasons (a: individual factor map, b: variable factor map). Variable factor map includes only the 15 most discriminant fatty acids.

The two‐way PERMANOVA revealed the presence of interactions between species and season factors (DF = 3 and 68, pseudo‐*F* = 2.717, *p*‐perm = .0041). A pairwise PERMANOVA showed that all lipid profiles of each factor combination were different from each other, except for winter and summer lipid profiles of *A. moerchi* and *M. discors*, respectively. Most of the variation in lipid profiles was observed on the first principal component, which discriminated *A. moerchi* from the other three species (Figure 4a). When the two principal components were combined, the lipid profiles of *M. truncata* were positioned between the lipid profiles of *A. moerchi* and those of *H. arctica* and *M. discors* (Figure 4a). Overall, *A. moerchi* was characterized by lower levels of PUFAs (especially 22:6ω3 and 20:5ω3) and higher levels of MUFAs (e.g., 20:1ω11; Figure 4b).

**FIGURE 5:**
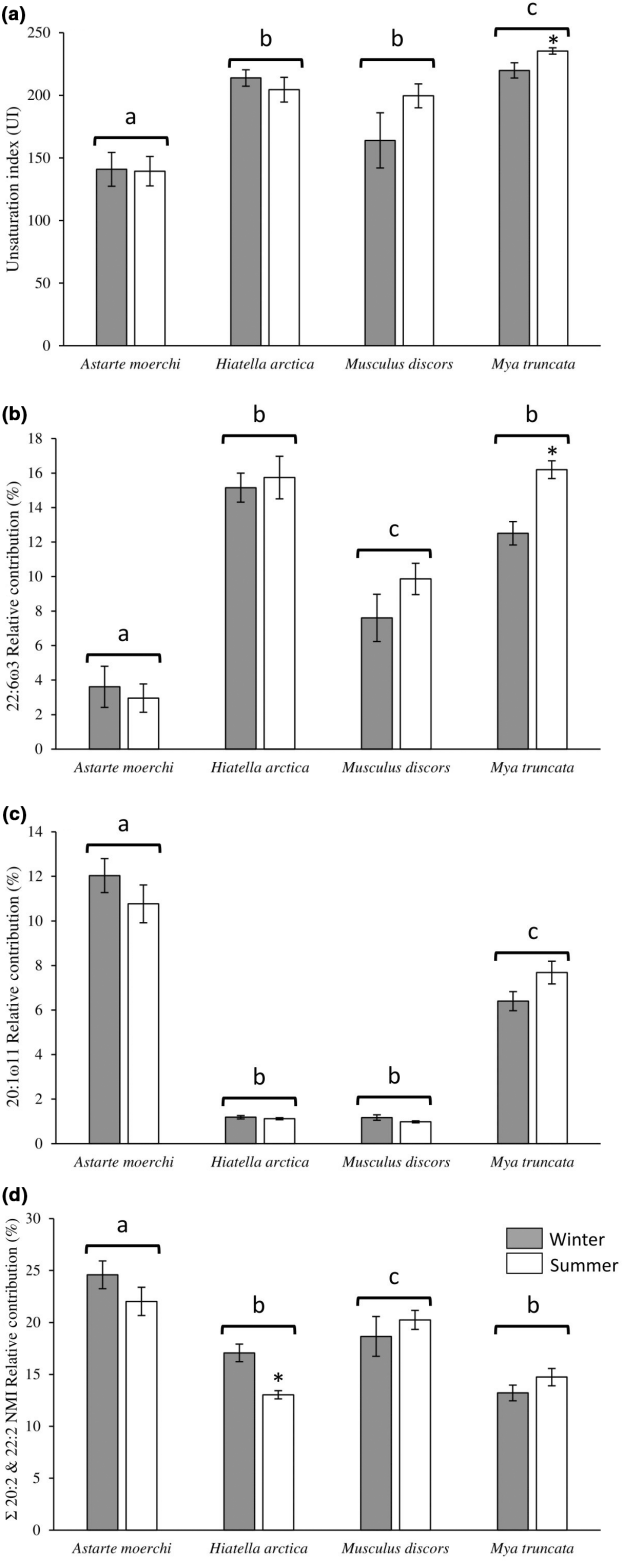
Seasonal and interspecific variations in unsaturation index (a), relative contributions of 22:6ω3 (b), 20:1ω11 (c) and sum of 20:2 and 22:2 NMI fatty acids (d) in polar lipids of four bivalve species. Data are mean ± Standard error of the mean (SEM). Letters indicate significant interspecific variations (winter and summer seasons combined) while asterisks indicate significant seasonal variations between lipid contents of a same species.

These differences in fatty acid profiles were confirmed by univariate two‐way PERMANOVAs realized separately for each specific fatty acid (Table S3). The UI and the sum of PUFAs displayed lower values for *A*. *moerchi* than for *H*. *arctica*, *M*. *discors*, and *M*. *truncata* (*p* < .05, Figure 5a, Table S3). These differences were mainly attributed to 22:6ω3, which displayed a twofold lower relative contribution in polar fatty acid profiles of *A. moerchi* than those of *M. discors* and a fourfold lower relative contribution than those observed for *H. arctica* and *M. truncata* (Figure 5b, Table S3). An opposite trend was observed for 20:1ω11, with *A. moerchi* showing the highest relative contribution (Figure 5c, Table S3). An intermediate relative contribution was observed for *M. truncata* while *H. arctica* and *M. discors* showed the lowest levels, approximately 10‐fold lower than those of *A. moerchi* (Figure 5c, Table S3). Finally, the sum of the 20:2 and 22:2 NMI showed higher values for *A*. *moerchi*, intermediate values for *M*. *discors*, and lower values for *H*. *arctica* and *M*. *truncata* (the latter characterized by the absence of 22:2) (Figure 5d, Table S3).

### 3.4 Specific fatty acid ratio from polar gill lipids to diet

**FIGURE 6:**
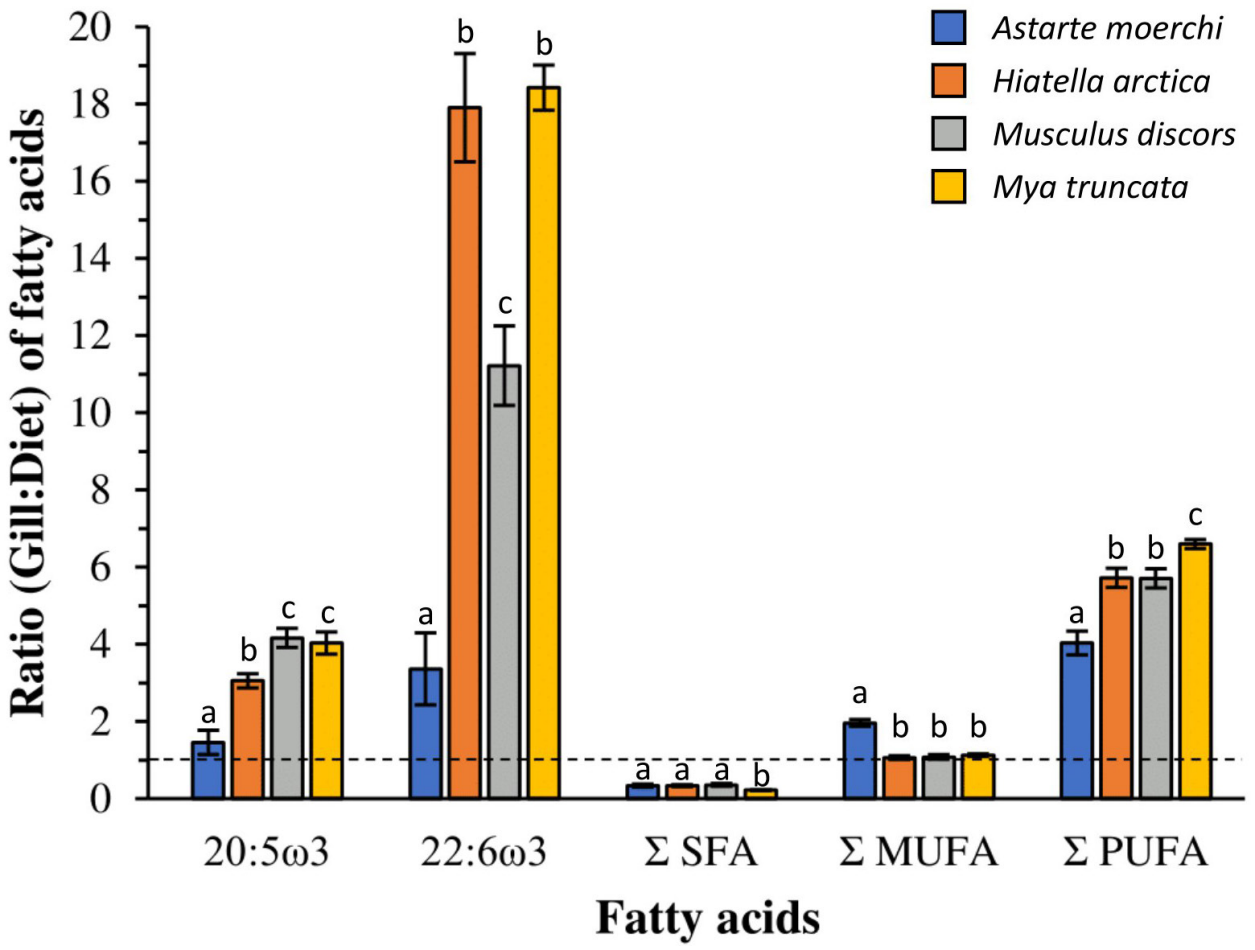
Ratio of polar fatty acids of gills to total fatty acids of particulate organic matter. The dashed line (ratio = 1) indicates equal amounts of fatty acids in gills and particulate organic matter. A Gill:Diet ratio for a specific fatty acid greater than 1 indicates selective retention of that specific fatty acid by the organism while a ratio lower than 1 reflects the absence of a physiological role for that fatty acid for the organism. Σ SFA, Σ MUGA, and Σ PUFA correspond to the sum of saturated, monounsaturated, and polyunsaturated fatty acids, respectively. The 20:5ω3 and 22:6ω3 represent the two fatty acids most involved in the metabolism and regulation of membrane fluidity in bivalves. Error bars represent the standard error of the mean (SEM).

For each specific fatty acid tested, the polar lipid fraction from gill to diet ratios showed variable values among the bivalves (Figure 6). Overall, *Astarte moerchi* showed lower Gill:Diet ratios for 20:5ω3 (Gill: Diet ratio = 1.5, DF = 3 and 37, pseudo‐*F* = 23.409, *p*‐perm = .0001) and 22:6ω3 (Gill: Diet ratio = 3.4, DF = 3 and 37, pseudo‐*F* = 42.27, *p*‐perm = .0001) than the other three species (Gill: Diet ratios >3 and 11 for 20:5ω3 and 22:6ω3, respectively). The same trend was observed for the sum of PUFAs as *A. moerchi* showed the lowest Gill:Diet ratios (DF = 3 and 37, pseudo‐*F* = 16.922, *p*‐perm = .0001). However, the variations among all species were less important, with 25% interspecies variation. The Gill:Diet ratio for MUFA remained around 1 for all species except *A*. *moerchi*, which had twice as much MUFA in its gills as in particulate organic matter (DF = 3 and 37, pseudo‐*F* = 49.049, *p*‐perm = .0001).

## 4 DISCUSSION

With climate change, Arctic coastal ecosystems will experience unprecedented environmental changes in sea ice extent and thickness, temperature, hydrological cycle, and stratification (Wassmann et al., 2020). All of these changes are likely to affect primary production and the quality of organic matter sources (e.g., PUFA content) in these low‐productivity ecosystems (Ardyna & Arrigo, 2020; Bridier et al., 2019; Michel et al., 2015). However, the lack of fundamental knowledge on the physiology of benthic invertebrates, which are essential links in Arctic marine food webs (Grebmeier, 2012), precludes a proper assessment of their vulnerability to these future qualitative and quantitative changes in the trophic environment. Considering the importance of PUFAs for the physiology of benthic invertebrates, especially for cold acclimation and maintenance of membrane fluidity (Hazel, 1995; Pernet et al., 2006; Sinensky, 1974), we believe it is essential to assess their fatty acid requirements to better evaluate their physiological vulnerability to future trophic changes. To address this knowledge gap, we investigated in this study the role of longevity on the fatty acid requirements of four bivalve species.

### 4.1 Feeding indicators

On both sampling occasions, *Musculus discors* showed at least 20% more lipid content in their digestive glands than the other species, suggesting a higher accumulation of food possibly related to increased feeding activity. Surprisingly, this species did not show significant seasonal differences suggesting a possible feeding behavior during the winter season. However, while such a behavior may have an energetic advantage (the species can assimilate lipid, protein, or carbohydrate compounds), this is not the case from a physiological point of view. EFA levels are probably too low at this time of year to be retained by the organisms to meet physiological requirements and allow the optimal control of membrane fluidity (and its effects on metabolism or cold acclimation). This was observed by Bridier et al. (2019), as the levels of EFAs (20:5ω3 and 22:6ω3) in particulate organic matter were so low in winter that they were below detection or quantification thresholds.

Seasonal variability was observed in *Astarte moerchi* and *Hiatella arctica*, which displayed an ~35% decrease in lipid content between winter and summer. The seasonal decrease in lipid content observed for *A. moerchi* and *H. arctica* may reflect (1) a strong allocation of energy reserves (i.e., egg production and spawning) between these two sampling periods toward reproduction (Amiraux et al., 2021; Birkely et al., 2003; Fokina et al., 2018; Pazos et al., 1997) and/or (2) high mobilization of energy reserves to meet the energetic needs of bivalves in the face of low food availability and/or interruption of bivalve feeding during winter (Ahn et al., 2003; Mayrand et al., 2017; McMeans et al., 2015). The mobilization of energy reserves for egg production usually involves a transfer of EFAs (especially 20:5ω3 and 22:6ω3) from the digestive glands to the gonads, resulting in a drastic decrease of these fatty acids in the digestive glands after reproductive investment (Leroy et al., 2013; Martínez‐Pita et al., 2012). The absence of such seasonal variations in our digestive gland lipid profiles suggests that no reproductive investment has occurred between our two sampling seasons. Although there is no information to our knowledge on the reproductive cycles of *A*. *moerchi* and *M*. *discors* in the literature, observations on *Hiatella arctica* and *Mya truncata* suggest that these two species spawn between June and October in Young Sound (Brandner et al., 2017; Veillard et al., 2023). Considering the absence of seasonal variations in digestive gland lipid profiles, it is possible that spawning occurred later (e.g., September or October), or was even completely suppressed this year if the bivalves had too low energy reserves to sustain this reproductive effort (Amiraux et al., 2021). Future changes in primary production (both in quantity and in quality) could therefore have dramatic consequences for the population dynamics of some bivalve species if the organisms do not have sufficient energy reserves to support the reproductive effort each year.

Seasonal differences seem more likely to reflect a mobilization of energy reserves during winter to meet the energy needs of bivalves. Considering the short period of primary production in Young Sound (restricted to June–September, Holding et al., 2019; Rysgaard et al., 1999) it is likely that *A. moerchi* and *H. arctica*, unlike *M. discors*, are unable to feed for most of the year. Although fjord‐shelf exchange may bring organic matter into the fjord from primary production occurring in the open ocean (which is ice‐free in May, Boone et al., 2017), the low quality of organic matter (i.e., high dominance of SFA) during this period suggests that this food source is primarily refractory (Bridier et al., 2019). Sea ice breakup occurred very late for Young Sound during our study (August 2, 2018, i.e., the latest observed breakup in a century, Greenland Ecosystem Monitoring database), as sea ice retreat usually occurs around mid‐July (Holding et al., 2019). Considering that primary production in Young Sound peaks 1–2 weeks prior to sea ice breakup (Rysgaard et al., 1999), the lipid content measured in our study is likely to be more depleted than would typically be observed at the same time in other years (e.g., Amiraux et al., 2021). However, these potential interannual differences remain small compared to many other Arctic sites with much more regular access to fresh organic matter throughout the year (e.g., Renaud et al., 2011). Our results thus highlight the unique trophic conditions in Young Sound where primary consumers have only 2–3 months to replenish their energy reserves for the winter period, as previously suggested by Sejr and Christensen (2007). In all bivalves collected, the dominant fatty acids in the neutral lipid fraction of the digestive gland were the 16:1ω7 and 20:5ω3 during the two seasons confirming their primary feeding on pelagic and/or benthic diatoms (Bridier et al., 2021). *M. discors* seems to be better adapted to ingest the available food, as its lipid content was systematically higher than that of other species without a significant decrease during winter conditions.

### 4.2 Physiological indicators

The fatty acid composition of polar lipids did not vary seasonally for *Astarte moerchi*, *Hiatella arctica*, and *Musculus discor*s. The absence of seasonal variation in the fatty acid composition of gills supports the homeoviscous adaptation theory (Hazel, 1995; Sinensky, 1974), which has been observed in marine bivalves (e.g., Hall et al., 2002; Parent et al., 2008; Pernet et al., 2007). To counteract the effect of seasonal temperature variations on cell membrane fluidity, marine bivalves undergo a significant lipid remodeling between winter and summer by increasing the proportion of MUFA to SFA (i.e., decreasing the SFA/MUFA ratio, Munro & Blier, 2012) and/or by increasing the proportion of PUFAs (especially in 20:5ω3 and 22:6ω3, Hall et al., 2002; Hazel, 1995; Pernet et al., 2007). However, all these studies were mainly conducted in subarctic or temperate or tropical systems, characterized by moderate or strong seasonal temperature variations (i.e., thermal amplitudes >6°C, Mathieu‐Resuge et al., 2020). The relatively stable temperatures in Young Sound (i.e., thermal amplitude <2.5°C at 17 m, Sejr et al., 2022) suggest that this temperature range is insufficient to induce a seasonal remodeling of lipid membranes. However, the fatty acid composition of polar lipids observed throughout the year likely reflects the acclimation of organisms to the low temperatures observed in the fjord (temperature < to 0.5°C throughout the year at 17 m, Sejr et al., 2022). The strong relative contributions of PUFAs observed in *H*. *arctica*, *M*. *discors*, and *Mya truncata* (i.e., >47%) are similar to those observed in other studies that also found an effect of cold temperature on the lipid composition of cell membranes of Arctic/sub‐Arctic bivalve species (Gaillard et al., 2015; Hacker Teper et al., 2022; Thyrring et al., 2017).

In contrast to the other three species, *A. moerchi* is characterized by much lower levels of PUFAs, especially 22:6ω3 (i.e., two to four times lower than the other species). Such low levels probably do not reflect a failure of *A. moerchi* to acclimate to cold temperatures but rather highlight the physiological characteristics of this species. According to the *membrane‐pacemaker theory of aging*, the differences in UI and relative contribution of 22:6ω3 observed between *A. moerchi*, *M. discors*, and *M. truncata* suggest that *A. moerchi* may have a lower metabolic rate than the other two species (Hulbert, 2005; Hulbert et al., 2007). Such a difference could reflect the longer longevity of *A. moerchi*, as long‐lived species typically have lower metabolic rates (associated with a lower production of reactive oxygen species) and modify the lipid composition of cell membranes by preferentially selecting fatty acids with the lowest number of double bonds to avoid peroxidation of membrane lipids (Blier et al., 2017). The higher levels of 20:2 and 22:2 NMI observed in gill lipid profiles of *A*. *moerchi* also support this hypothesis, as these fatty acids are known to be more resistant to oxidative processes than other PUFAs (Barnathan, 2009). A previous study highlighted the ability of *Astarte borealis* to significantly reduce its metabolism under anoxic conditions to less than 0.5% of its aerobic rate (Oeschger, 1990). One might expect that such a metabolic strategy, known as metabolic rate depression (Abele et al., 2009), might also occur in *A*. *moerchi* to cope with other stressful environmental conditions such as low temperature and/or poor trophic conditions, as is the case in Young Sound.

Surprisingly, *H*. *arctica* considered with long lifespan (i.e., ML ≈ 120 years, Sejr et al., 2002) showed similar lipid dynamics in their cell membranes of gills than short‐lived species (i.e., similar UI and PUFA level). Thus, *H. arctica* could have metabolic rate at similar levels than *M. truncata* and *M. discors* and probably different from the potentially reduced metabolic rate of long‐lived *A. moerchi* showing the lowest levels of PUFAs and 22:6ω3 in their gill membranes. The digestive glands of *H. arctica* also show the greatest seasonal variation in lipid content suggesting that this species may mobilize its energy reserves during winter to potentially cover the metabolic costs. Such results may indicate that *H. arctica* does not enter a metabolically depressed state like *A. moerchi* might when environmental conditions are stressful.

One hypothesis can be suggested to explain why two long‐lived species (i.e., *A. moerchi* and *H. arctica*) adopt different fatty acid dynamics in gill membranes during winter. It may be that cold acclimation and metabolism act in opposite directions in regulating the fatty acid composition of cell membranes. If bivalves regulate the cell membrane fluidity by increasing the proportion of PUFAs and especially 22:6ω3 to adapt to cold (i.e., homeoviscous adaptation, e.g., Pernet et al., 2006), such a lipid remodeling would also inevitably lead to an increase in metabolic rate (according to the membrane pacemaker theory of metabolism, Hulbert, 2005). We can therefore speculate that *A*. *moerchi* and *H*. *arctica* use different strategies to acclimate to cold temperature. Homeoviscous adaptation does not necessarily involve a mere increase in the proportion of PUFAs but may also involve additional mechanisms such as an increase in the proportion of MUFAs, a decrease in cholesterol content, or an elongation of fatty acid carbon chains (Hazel, 1995; Pernet et al., 2006; Sinensky, 1974). *H. arctica* may mainly regulate the fluidity of cell membranes by increasing the proportion of PUFAs while *A. moerchi* may favor some of these additional mechanisms to acclimate to cold temperature without affecting the metabolic rate. The low relative contribution of PUFAs in *A. moerchi* cell membranes combined with the high relative contribution of de novo synthetized MUFAs with long carbon chains (e.g., 20:1ω11) or NMI fatty acids (which also ensure good membrane fluidity with less unsaturation than PUFAs, Barnathan, 2009; Kraffe et al., 2004) seems to confirm this hypothesis. Therefore, maintaining high levels of PUFAs in cell membranes for cold acclimation would inevitably lead to an increase in metabolism and could ultimately result in a mismatch between the high energy needs of *H. arctica* and the limited lipid reserves of long‐lived species. This hypothesis seems to be confirmed by the seasonal dynamics of digestive gland lipid content, since *H. arctica* has both the lowest total lipid content and the highest seasonal variation among the four species. On the other hand, *A. moerchi* might maintain a reduced metabolism and thus better match the size of its energy reserves with the rate of their depletion by favoring other cold acclimation mechanisms such as a decrease in the SFA/MUFA ratio (Munro & Blier, 2012) or decrease in the polar lipid/sterol ratio (i.e., an increase in the proportion of sterol compared to the proportion of polar lipids, Parent et al., 2008). Such contrasting physiological strategies for regulating membrane fluidity between two long‐lived species may reflect differences in their geographic distribution. While *A*. *moerchi* is restricted to Arctic waters (De Cesare et al., 2017; Olivier et al., 2020; Petersen, 2001), *H*. *arctica* has a broad geographic distribution from temperate latitudes (e.g., Azores, Mediterranean Sea) to the Arctic Ocean (Cardigos et al., 2006; Hunter, 1949; Manousis, 2021). The biogeography of *H*. *arctica* may therefore imply that this species is not as well adapted as *A*. *moerchi* to cold Arctic environments. It is possible that biogeography also explains some of the differences observed between the lipid profiles of *A*. *moerchi* and those of *M*. *discors* and *M*. *truncata*, since the latter two species are also characterized by a wider geographic distribution than *A*. *moerchi* (Günther & Fedyakov, 2000; Petersen, 1978). We believe that the role of biogeography in these interspecific physiological differences is probably small compared to the role of longevity, since longevity is known to be the main factor controlling the fatty acid composition of cell membranes (Munro & Blier, 2015). However, it would be interesting for future studies to attempt to separate the confounding effects of biogeography and longevity by selecting circumpolar, boreal and/or cosmopolitan species with different longevities within each geographic distribution.

### 4.3 Selective retention of EFAs

The variation in Gill:Diet ratios among the four species reflects the trends observed in the fatty acid composition of polar lipids (see previous section). All bivalves have a Gill:Diet ratio for total PFAs (Σ PUFA) higher than 1, reflecting the need for these species to selectively retain PUFAs from their diet to maintain the integrity of their cell membranes (Gendron et al., 2013; Mejri et al., 2021). Such incorporation of PUFAs into membrane lipids may be partly explained by the physiological requirements of bivalves for highly unsaturated fatty acids with long carbon chains (e.g., 20:5ω3 and 22:6ω3), which are crucial for maintaining cell membrane fluidity in the face of cold (i.e., *homeoviscous adaptation*, Pernet et al., 2006). However, as feeding regime was dominated by 20:5 ω3 from diatoms, their selective retention is largely lower than that observed for 22:6ω3, which is poorly represented in the diet. Some variation in Gill:Diet ratios is also observed among the four bivalve species. *A. moerchi* has significantly lower ratios for PUFAs, especially for EFAs such as 20:5ω3 and 22:6ω3 which are two to six times less selectively retained in *A*. *moerchi* cell membranes than in the other three species. As discussed above, these differences probably highlight the physiological characteristics of *A. moerchi* and in particular its long lifespan, which may be related to the lower proportion of PUFAs in lipid membranes to reduce oxidative stress (Blier et al., 2017) and maintain a slower metabolism (Hulbert, 2005; Hulbert et al., 2007). In contrast to the other species, the Σ MUFA Gill:Diet ratio is twice as high in *A. moerchi* (Gill: Diet ratio = 2.0), mainly due to 20:1ω11 (representing about one third of the total relative contribution of MUFAs). However, this probably does not reflect a selective retention of MUFAs by *A. moerchi* but rather reflects de novo synthesis of 20:1ω11 to adjust membrane fluidity (Kraffe et al., 2004; Mathieu‐Resuge et al., 2020).

### 4.4 Need for new knowledge of benthic invertebrate physiology on a pan‐Arctic scale

The results discussed above provide some insight on the vulnerability of benthic invertebrates to change in Arctic ecosystems. However, it should be kept in mind that our results come from the study of a single Arctic region and a single study site (i.e., Young Sound, North‐East Greenland). Therefore, it is important that a larger number of future studies focus on acquiring new fundamental information on the physiology of benthic invertebrates in order to accurately assess their vulnerability to climate change (Renaud et al., 2015, 2019). The comparison of several stations distributed along a trophic gradient (e.g., a gradient extending from the inner to the outer part of a fjord, Bridier et al., 2019) could help to better assess the degree of importance of interspecies physiological differences in the resilience/vulnerability of invertebrates to future trophic changes. The acquisition of new data on the physiology of benthic invertebrates in different Arctic regions would also allow a better understanding of how these physiological acclimations may vary locally according to the local environmental characteristics (latitude, depth, distance from shore, etc.). Only the synthesis of these future studies will provide a comprehensive understanding of the vulnerability of benthic invertebrates to future trophic changes on a pan‐Arctic scale.

## 5 CONCLUSIONS AND OUTLOOK

**FIGURE 7:**
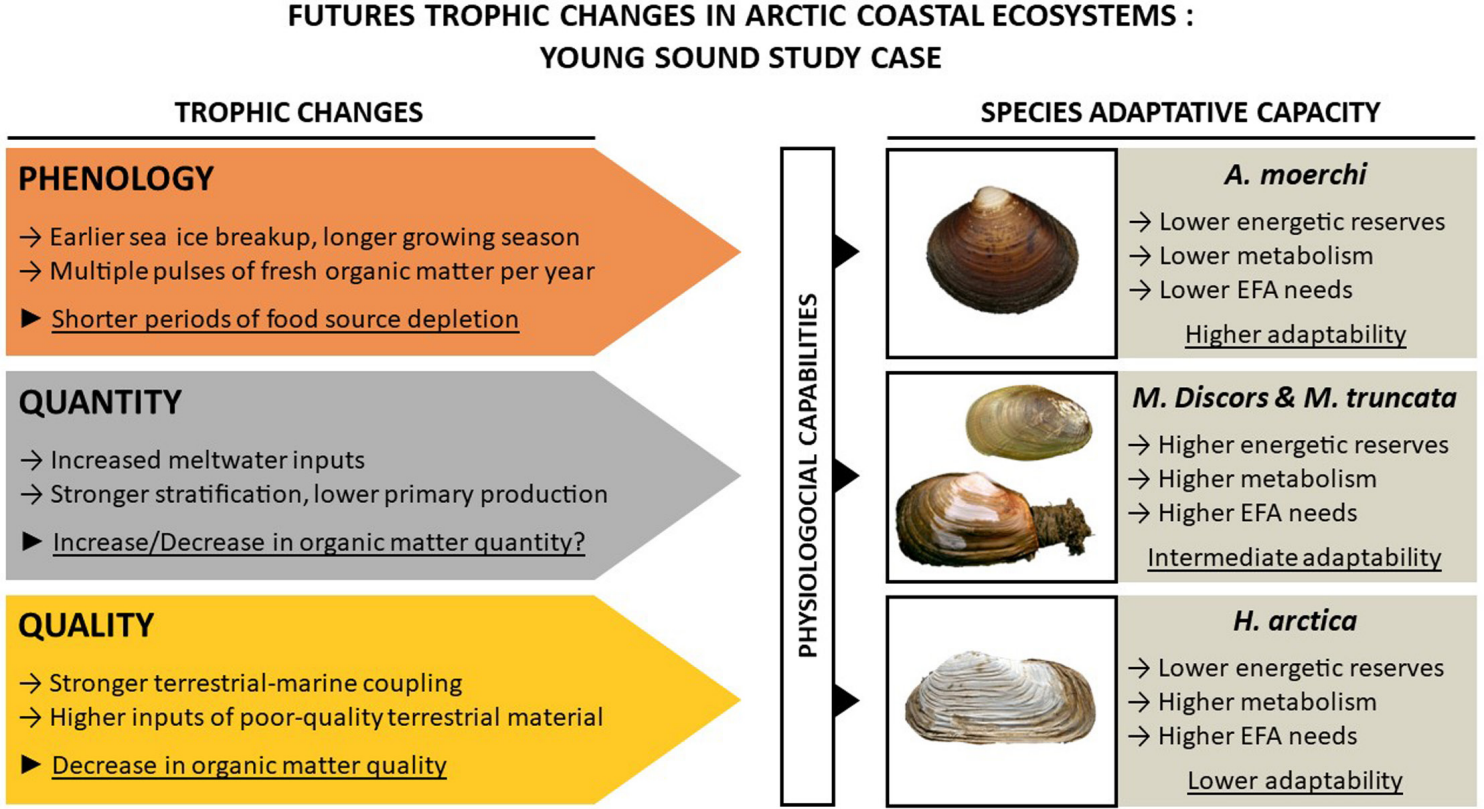
A schematic attempting to summarize future trophic constraints in Young Sound (in terms of primary producer phenology and organic matter quantity/quality) with the expected physiological capabilities of the four studied bivalve species to cope with these future trophic changes.

Arctic coasts will experience multiple environmental changes related to cryosphere loss that will subsequently affect overall ecosystem functioning. Increased glacier melt and meltwater inputs to coastal ecosystems are likely to change the phenology of primary producers, modify the ecosystem productivity, and alter the nutritional quality of organic matter sources available to primary consumers. New fundamental knowledge about the physiology and nutritional needs of Arctic benthic invertebrates is therefore essential if we want to properly assess their vulnerability to future environmental changes in coastal ecosystems. Fatty acids are a powerful tool that can provide information on the spatiotemporal changes in the source, quality, and quantity of food sources, as well as reflect physiological responses to environmental stress. The strong disparities observed among the four bivalve species in their fatty acid dynamics and remodeling in relation to their nutritional needs suggest that not all benthic invertebrates will be equally vulnerable to future trophic changes (Figure 7). Species characterized by long life expectancy and evidence of low metabolism (e.g., *A. moerchi*) are likely to be best adapted to future trophic conditions, as their low EFA requirements (e.g., 20:5ω3 and 22:6ω3) may help them to cope with a decrease in organic matter quality. In contrast, species with a shorter life expectancy (e.g., *Musculus discors* and *Mya truncata*) may have a higher energetic turnover and thus a higher demand for EFAs. On the other hand, more regular inputs of fresh organic matter (due to longer ice‐free period and growing season for phytoplankton) may help these species to meet their high metabolic needs during winter (due to the absence of winter dormancy) without depleting their energy reserves. Finally, species characterized by long life expectancy and evidence of high metabolic rate (e.g., *Hiatella arctica*) may be the least adapted to these future trophic conditions because they have both high EFA and energy requirements (due to their high metabolic rates) but limited energy reserves.

These interspecific differences in vulnerability to future environmental change may have broader consequences for the functioning of Arctic coastal ecosystems. Invertebrates with short life spans and high metabolic rates also have higher growth and productivity, and therefore represent optimal carbon transfer pathways within Arctic food webs. This is the case for *M*. *truncata*, which is an essential link in the transfer of organic matter produced by phytoplankton or microphytobenthos to higher trophic levels (e.g., walruses and eiders, Born et al., 2003; Frimer, 1997). Conversely, species with long life spans and low metabolic rates (e.g., *A*. *moerchi*) have lower growth and productivity, and therefore contribute less to carbon transfers within trophic webs. Consequently, the increased vulnerability of high metabolic rate species to future trophic changes may have further consequences for coastal Arctic food webs by reducing carbon flows to higher trophic levels.

## AUTHOR CONTRIBUTIONS

**Guillaume Bridier:** Conceptualization (equal); formal analysis (equal); investigation (equal); methodology (equal); software (equal); visualization (equal); writing – original draft (equal). **Frédéric Olivier:** Conceptualization (equal); funding acquisition (equal); project administration (equal); supervision (equal); writing – review and editing (equal). **Jacques Grall:** Conceptualization (equal); funding acquisition (equal); project administration (equal); supervision (equal); writing – review and editing (equal). **Laurent Chauvaud:** Conceptualization (equal); funding acquisition (equal); project administration (equal); supervision (equal); writing – review and editing (equal). **Mikael K. Sejr:** Conceptualization (equal); funding acquisition (equal); project administration (equal); writing – review and editing (equal). **Réjean Tremblay:** Conceptualization (equal); funding acquisition (equal); project administration (equal); resources (equal); supervision (equal); writing – review and editing (equal).

## Supporting information


Tables S1–S3.

## Data Availability

The data that support the findings of this study are available in the Supporting Information. Authors are available upon request for additional information on data analyses.
