# Supplementary material for: Seasonal lipid dynamics of four Arctic bivalves: Implications for their physiological capacities to cope with future changes in coastal ecosystems
Source: Ecol Evol. 2023 Nov 2;13(11):e10691. doi: 10.1002/ece3.10691 (PMC10620577; doi:10.1002/ece3.10691)
Supplement: Supplementary file 1 — Tables S1–S3. [file ECE3-13-e10691-s001.docx]

*The following supplement accompanies the article*

**Seasonal lipid dynamics of four Arctic bivalves: implications for their physiological capacities to cope with future changes in coastal ecosystems**

**Guillaume Bridier*, Frédéric Olivier, Jacques Grall, Laurent Chauvaud, Mikael K. Sejr, Réjean Tremblay**

*Corresponding author: guillaume.bridier@live.ie

Table S1: Mass of total lipids, wet weight of digestive glands, total lipid content of digestive glands (DG) and shell sizes (length, width, height) of *Astarte* *moerchi*, *Hiatella* *arctica*, *Mya* *truncata* and *Musculus* *discors* collected in Winter and Summer. NA: Not Available. Missing values (NA) are related to two situations: 1) the amount of lipid or the mass of the digestive gland were too small to estimate lipid concentrations with reasonable accuracy, 2) the bivalve shells broke during dissection.

Table S2: Neutral lipid fatty acid composition of *Astarte moerchi*, *Hiatella arctica*, *Mya truncata* and *Musculus discors* digestive glands in Winter (Mai) and Summer (August). Values correspond to mean percentages with standard errors of the mean in brackets. SFA, MUFA, PUFA and EFA refer to Saturated, MonoUnsaturated, PolyUnsaturated and Essential Fatty Acids, respectively. EPA: Eicosapentaenoic acid (i.e. 20:5ω3), DHA: Docosahexaenoic acid (i.e. 22:6ω3), TMTD: Trimethyltridecanoic acid, UI: Unsaturation Index. Fatty acid percentages < 0.2 % in all samples are not shown. Asterisks and percentages in bold represent significantly different fatty acid levels between two seasons in a same species (p < 0.05). Different letters indicate significantly different fatty acid levels between species with winter and summer seasons combined (p-values are shown in the last column on the right).

Table S3: Polar lipid fatty acid composition of *Astarte moerchi*, *Hiatella arctica*, *Mya truncata* and *Musculus discors* gills in Winter (May) and Summer (August). Values correspond to mean percentages with standard errors of the mean in brackets. SFA, MUFA, PUFA and EFA refer to Saturated, MonoUnsaturated, PolyUnsaturated and Essential Fatty Acids, respectively. EPA: Eicosapentaenoic acid (i.e. 20:5ω3), DHA: Docosahexaenoic acid (i.e. 22:6ω3), TMTD: Trimethyltridecanoic acid, UI: Unsaturation Index. Fatty acid percentages < 0.2 % in all samples are not shown. Asterisks and percentages in bold represent significantly different fatty acid levels between two seasons in a same species (p < 0.05). Different letters indicate significantly different fatty acid levels between species with winter and summer seasons combined (p-values are shown in the last column on the right).

**Table S1**

| **Species** | **Season** | **Total Lipid (mg)** | **GD (g)** | **[Total Lipids GD] (mg/g)** | **Length** | **Width** | **Height** |
| --- | --- | --- | --- | --- | --- | --- | --- |
| *Astarte moerchi* | Winter | 2,49 | 0,04 | 59,46 | 27,05 | 21,17 | 10,40 |
|  |  | NA | NA | NA | 22,47 | 18,88 | 7,38 |
|  |  | NA | NA | NA | 42,37 | 32,73 | 16,09 |
|  |  | 1,43 | 0,04 | 35,73 | 30,88 | 23,71 | 11,62 |
|  |  | 0,31 | 0,01 | 51,22 | 30,04 | 23,09 | 11,91 |
|  |  | NA | NA | NA | 26,51 | 19,64 | 9,66 |
|  |  | 4,27 | 0,13 | 33,24 | 36,75 | 28,72 | 13,61 |
|  |  | 3,24 | 0,09 | 34,94 | NA | NA | NA |
|  |  | 2,74 | 0,07 | 41,27 | 27,85 | 21,13 | 8,74 |
|  |  | 5,66 | 0,17 | 34,28 | 32,98 | 25,75 | 13,66 |
|  |  | 2,96 | 0,08 | 35,87 | 29,01 | 22,14 | 10,76 |
|  |  | 1,48 | 0,03 | 53,82 | NA | NA | NA |
|  | Summer | NA | NA | NA | 35,50 | 28,50 | 13,59 |
|  |  | 2,10 | 0,08 | 26,21 | 32,89 | 26,31 | 12,54 |
|  |  | 0,80 | 0,03 | 23,39 | 31,13 | 23,43 | 10,75 |
|  |  | 1,41 | 0,05 | 27,45 | 36,42 | 28,26 | 13,65 |
|  |  | 2,11 | 0,05 | 42,64 | 31,29 | 24,51 | 11,73 |
|  |  | NA | NA | NA | 39,27 | 31,91 | 13,12 |
|  |  | 3,96 | 0,11 | 36,52 | NA | NA | NA |
|  |  | 4,95 | 0,13 | 38,67 | 40,60 | 30,70 | 15,79 |
|  |  | 0,81 | 0,04 | 20,90 | 40,6 | 30,7 | 15,79 |
|  |  | 1,96 | 0,09 | 22,99 | 34,56 | 26,33 | 12,37 |
|  |  | 5,25 | 0,13 | 38,94 | 33,79 | 27,57 | 12,07 |
| *Hiatella arctica* | Winter | 3,07 | 0,06 | 47,55 | 46,36 | 21,07 | 21,97 |
|  |  | 2,95 | 0,10 | 28,68 | 39,92 | 19,37 | 20,57 |
|  |  | 1,31 | 0,05 | 24,86 | 39,64 | 20,31 | 20,33 |
|  |  | NA | NA | NA | 42,13 | 18,27 | 17,00 |
|  |  | 4,46 | 0,05 | 89,99 | 44,66 | 21,53 | 16,59 |
|  |  | 1,43 | 0,03 | 41,41 | 36,55 | 19,81 | 18,33 |
|  |  | NA | NA | NA | 41,97 | 21,05 | 17,67 |
|  |  | NA | NA | NA | 43,86 | 18,98 | 17,77 |
|  |  | 2,91 | 0,06 | 52,74 | 39,80 | 19,22 | 17,70 |
|  | Summer | 1,66 | 0,07 | 24,11 | 37,59 | 16,58 | 18,52 |
|  |  | 1,93 | 0,08 | 23,67 | NA | NA | NA |
|  |  | 1,17 | 0,09 | 13,63 | 37,65 | 18,89 | 16,15 |
|  |  | 2,82 | 0,08 | 36,94 | 41,08 | 16,97 | 20,65 |
|  |  | 3,42 | 0,18 | 18,70 | 43,90 | 20,82 | 18,06 |
|  |  | 2,86 | 0,10 | 28,14 | 40,01 | 18,26 | 19,21 |
|  |  | 1,91 | 0,09 | 22,47 | 38,35 | 18,65 | 16,79 |
|  |  | 0,90 | 0,04 | 25,55 | 34,98 | 17,30 | 16,34 |
|  |  | 2,08 | 0,06 | 35,50 | 32,69 | 18,16 | 14,48 |
|  |  | 0,98 | 0,05 | 18,96 | 31,89 | 16,26 | 14,56 |
|  |  | 2,68 | 0,15 | 17,73 | 39,01 | 18,99 | 15,94 |
| *Mya truncata* | Winter | 14,40 | 0,38 | 37,44 | NA | NA | NA |
|  |  | 20,47 | 0,34 | 60,72 | 60,96 | 35,39 | 24,41 |
|  |  | NA | NA | NA | 45,04 | 35,35 | 20,08 |
|  |  | 8,44 | 0,38 | 21,97 | 50,68 | 38,48 | 26,69 |
|  |  | NA | NA | NA | 42,54 | 29,79 | 20,59 |
|  |  | NA | NA | NA | 43,84 | 33,25 | 24,15 |
|  |  | NA | NA | NA | 40,84 | 28,33 | 16,79 |
|  | Summer | 14,99 | 0,22 | 67,65 | 41,57 | 27,19 | 17,50 |
|  |  | 5,79 | 0,21 | 27,60 | NA | NA | NA |
|  |  | 17,78 | 0,39 | 46,06 | 41,10 | 28,26 | 18,39 |
|  |  | NA | NA | NA | 40,17 | 30,03 | 20,88 |
|  |  | 15,11 | 0,43 | 35,50 | 42,81 | 30,28 | 18,63 |
|  |  | 40,40 | 0,59 | 68,15 | 50,20 | 37,36 | 23,60 |
|  |  | NA | NA | NA | 36,37 | 26,57 | 17,03 |
|  |  | NA | NA | NA | 36,05 | 26,89 | 17,28 |
| *Musculus discors* | Winter | 3,41 | 0,05 | 66,57 | NA | NA | NA |
|  |  | NA | NA | NA | 22,21 | 14,84 | 9,96 |
|  |  | 1,46 | 0,02 | 65,36 | 25,47 | 15,97 | 12,32 |
|  |  | 1,57 | 0,04 | 39,10 | 24,60 | 16,12 | 10,68 |
|  |  | 4,96 | 0,05 | 97,23 | 28,86 | 19,03 | 11,95 |
|  |  | 0,95 | 0,02 | 51,03 | 18,32 | 12,21 | 7,81 |
|  |  | 0,72 | 0,03 | 26,13 | 24,66 | 16,02 | 11,36 |
|  | Summer | NA | NA | NA | 30,88 | 20,09 | 12,80 |
|  |  | 1,61 | 0,08 | 21,11 | 26,59 | 17,52 | 10,79 |
|  |  | NA | NA | NA | 24,40 | 15,39 | 9,94 |
|  |  | 3,55 | 0,08 | 45,45 | 26,29 | 14,44 | 8,71 |
|  |  | 1,49 | 0,05 | 32,24 | NA | NA | NA |
|  |  | NA | NA | NA | 23,16 | 15,10 | 9,80 |
|  |  | NA | NA | NA | 25,12 | 16,44 | 10,76 |
|  |  | 7,76 | 0,13 | 60,55 | 24,80 | 16,36 | 10,84 |
|  |  | 3,17 | 0,06 | 57,20 | 27,09 | 17,41 | 12,97 |
|  |  | NA | NA | NA | 21,07 | 13,29 | 9,32 |

**Table S2**

|  | *Astarte* *moerchi* | | *Hiatella* *arctica* | | *Mya* *truncata* | | *Musculus* *discors* | | p-perm |
| --- | --- | --- | --- | --- | --- | --- | --- | --- | --- |
|  | Summer | Winter | Summer | Winter | Summer | Winter | Summer | Winter |  |
| 12:0 | tr | tr (a) | nd | nd (b) | tr | tr (a) | 0.5 (0.1) | 0.3 (0.1) (c) | **0.0001** |
| 14:0 | **2.8 (0.3) (*)** | **3.6 (0.2) (a)** | 3.7 (0.3) | 3.5 (0.1) (a) | 5.1 (0.3) | 3.5 (0.3) (a) | 6.4 (0.2) | 5.3 (0.4) (b) | **0.0001** |
| 15:0 | 0.3 (0.0) | 0.3 (0.0) (a) | 0.4 (0.0) | 0.3 (0.0) (a) | 0.2 (0.0) | 0.2 (0.1) (b) | 0.3 (0.0) | 0.3 (0.0) (a) | **0.0005** |
| 16:0 | 12.1 (0.9) | 12.6 (0.7) (ab) | 14.1 (0.6) | 13.5 (0.8) (a) | 11.5 (0.9) | 10 (1.6) (c) | 12.1 (0.6) | 12.5 (0.7) (ab) | **0.0227** |
| 17:0 | 0.2 (0.0) | 0.2 (0.0) (a) | 0.3 (0.0) | 0.3 (0.0) (b) | tr | 0.2 (0.0) (c) | tr | tr (c) | **0.0001** |
| 18:0 | 4.1 (1.3) | 3.2 (1.0) (ab) | **6.1 (0.8) (*)** | **3.8 (0.4) (a)** | 1.5 (0.1) | 2.8 (0.5) (ab) | 1.7 (0.4) | 2.3 (0.7) (b) | **0.0034** |
| Σ SFA | 19.7 (2.2) | 19.9 (1.4) (ac) | **24.6 (0.8) (*)** | **21.6 (0.7) (b)** | 18.7 (0.7) | 16.8 (1.3) (ac) | 21.3 (0.6) | 21.4 (1.1) (ab) | **0.0002** |
| 14:1ω | nd | nd | nd | nd | nd | nd | 0.3 (0.1) | 0.4 (0.0) | - |
| 16:1ω7 | 16.5 (1.6) | 20.1 (1.2) (a) | 13.7 (1) | 14.4 (0.6) (b) | 23.3 (0.7) | 20.2 (2.2) (a) | 24.6 (1.0) | 20.3 (1.5) (a) | **0.0001** |
| 18:1ω7 | 5.5 (0.5) | 6.0 (0.4) (a) | **6.8 (0.4) (*)** | **10.6 (0.4) (b)** | **5.1 (0.1) (*)** | **7.8 (0.9) (a)** | 1.1 (0.0) | 1.5 (0.2) (c) | **0.0001** |
| 18:1ω9 | 2.2 (0.2) | 2.7 (0.2) (a) | 1.4 (0.2) | 1.2 (0.2) (b) | **1.0 (0.1) (*)** | **2.0 (0.3) (b)** | 1.3 (0.0) | 1.6 (0.2) (b) | **0.0001** |
| 18:1ω11 | 0.5 (0.1) | 0.6 (0.2) (a) | **1.1 (0.2) (*)** | **0.6 (0.1) (b)** | **0.3 (0.0) (*)** | **1.6 (0.5) (c)** | 0.3 (0.1) | 0.4 (0.2) (b) | **0.0276** |
| 20:1ω7 | 3.8 (0.4) | 3.9 (0.5) (a) | **3.6 (0.3) (*)** | **5.1 (0.4) (b)** | 1.6 (0.1) | 3.7 (0.8) (c) | 2.6 (0.2) | 3.0 (0.4) (abc) | **0.0103** |
| 20:1ω9 | 1.1 (0.2) | 1.3 (0.1) | 1.4 (0.1) | 1.3 (0.2) | 0.6 (0.1) | 1.4 (0.4) | 1.3 (0.1) | 1.7 (0.2) | 0.6242 |
| 20:1ω11 | 2.7 (0.5) | 2.3 (0.3) (a) | 0.4 (0.0) | 0.4 (0.0) (b) | tr | 0.7 (0.3) (b) | 0.3 (0.0) | 0.6 (0.2) (b) | **0.0001** |
| 22:1ω9 | 0.4 (0.1) | 0.3 (0.0) (a) | 0.7 (0.1) | 0.5 (0.1) (b) | tr | 0.3 (0.1) (c) | 0.2 (0.0) | 0.3 (0.1) (ac) | **0.0001** |
| 22:1ω11 | 0.3 (0.1) | tr (a) | 0.4 (0.1) | 0.4 (0.1) (b) | tr | tr (c) | tr | tr (c) | **0.0001** |
| 24:1ω9 | 0.6 (0.2) | 0.8 (0.2) (a) | **1.4 (0.2) (*)** | **0.8 (0.2) (b)** | 0.5 (0.1) | 0.7 (0.2) (a) | 0.2 (0.1) | nd (c) | **0.0001** |
| Σ MUFA | 33.7 (1.5) | 38.3 (1.7) (a) | **31 (0.7) (*)** | **35.3 (0.9) (ab)** | 33 (0.9) | 38.4 (2.7) (a) | 32.5 (0.8) | 30.2 (1.1) (b) | **0.0032** |
| 16:2ω | 0.4 (0.1) | 0.4 (0.1) (a) | tr | tr (b) | 0.4 (0.1) | tr (c) | 0.7 (0.1) | 0.7 (0.0) (d) | **0.0001** |
| 18:2ω4 | 0.4 (0.1) | 0.3 (0.0) (a) | 0.8 (0.1) | 0.4 (0.1) (b) | 0.4 (0.0) | 0.5 (0.1) (c) | 0.3 (0.0) | 0.4 (0.1) (a) | **0.0080** |
| 18:2ω6 | 2.1 (0.7) | 1.9 (0.1) (a) | 0.6 (0.1) | 0.5 (0.0) (b) | 0.5 (0.0) | 0.5 (0.0) (b) | 0.7 (0.0) | 0.9 (0.1) (b) | **0.0001** |
| 18:3ω3 | 0.5 (0.1) | 0.6 (0.0) (a) | 0.7 (0.0) | 0.7 (0.0) (b) | 0.4 (0.0) | 0.2 (0.1) (c) | 0.8 (0.0) | 0.9 (0.1) (d) | **0.0001** |
| 18:3ω6 | 0.2 (0.0) | 0.2 (0.0) (ab) | 0.5 (0.1) | 0.2 (0.0) (a) | 0.2 (0.0) | 0.3 (0.1) (ab) | tr | tr (b) | **0.0034** |
| 18:4ω3 | 1.1 (0.2) | 1.6 (0.1) (a) | 2.9 (0.2) | 1.8 (0.1) (b) | **2.3 (0.1) (*)** | **1.1 (0.1) (a)** | 3.9 (0.2) | 3.2 (0.2) (c) | **0.0001** |
| 20:2 | **0.5 (0.0) (*)** | **0.7 (0.0) (a)** | 1.1 (0.1) | 0.8 (0.1) (b) | **0.4 (0.0) (*)** | **0.6 (0.1) (ac)** | 0.4 (0.0) | 0.6 (0.1) (c) | **0.0001** |
| 20:2ω | 2.5 (0.9) | 1.7 (0.3) (ab) | 1.1 (0.1) | 2.4 (0.2) (a) | 0.4 (0.0) | 0.9 (0.1) (b) | 1.6 (0.1) | 2.3 (0.4) (a) | 0.0607 |
| 20:3ω6 | 0.3 (0.0) | 0.4 (0.1) (a) | 0.4 (0.1) | 0.2 (0.0) (a) | tr | tr (b) | tr | 0.2 (0.0) (ab) | **0.0095** |
| 20:4ω6 | 1.4 (0.2) | 1.0 (0.1) (a) | 1.0 (0.1) | 0.7 (0.1) (ab) | 0.8 (0.1) | 1.3 (0.5) (a) | 0.6 (0.1) | 0.7 (0.1) (b) | **0.0023** |
| 20:5ω3 | 26.4 (2.8) | 24.6 (3.0) (a) | 24.4 (1.1) | 26.1 (1.4) (a) | 37 (0.5) | 33.8 (2.7) (b) | 29.1 (0.5) | 26.7 (2.1) (a) | **0.0006** |
| 22:2ω | 0.8 (0.1) | 0.7 (0.1) (a) | 0.8 (0.1) | 1.0 (0.1) (a) | tr | tr (b) | 0.4 (0.1) | 1.2 (0.2) (a) | **0.0001** |
| 22:2ωbis | 1.3 (0.2) | 1.6 (0.2) | nd | nd | nd | nd | nd | nd | - |
| 22:6ω3 | 5.0 (1.1) | 3.6 (0.5) (ab) | 5.6 (0.5) | 4.8 (0.3) (a) | 3.4 (0.2) | 2.7 (0.5) (b) | 3.4 (0.3) | 4.4 (0.3) (ab) | **0.0459** |
| Σ PUFA | 45.4 (3) | 40.5 (3) (abc) | 43 (0.7) | 41.7 (1.3) (a) | 47.6 (0.4) | 44 (2.7) (abc) | 44.9 (0.5) | 46.6 (0.9) (c) | **0.0493** |
| 16.0iso | tr | tr (a) | 0.5 (0.1) | 0.3 (0.0) (b) | tr | tr (c) | tr | 0.2 (0.1) (abc) | **0.0039** |
| 17.0anteiso | 0.2 (0.0) | 0.2 (0.0) (a) | tr | 0.2 (0.0) (a) | tr | tr (b) | tr | tr (abc) | **0.0001** |
| Σ BrFA | 0.7 (0.0) | 0.6 (0.1) (a) | 0.9 (0.1) | 0.8 (0.1) (a) | 0.4 (0.0) | 0.3 (0.1) (b) | 0.4 (0.1) | 0.7 (0.2) (ab) | **0.0008** |
| TMTD | 0.5 (0.0) | 0.7 (0.1) (a) | 0.5 (0.1) | 0.6 (0.0) (a) | 0.3 (0.0) | 0.5 (0.2) (b) | 0.8 (0.1) | 1.1 (0.1) (c) | **0.0001** |
| Σ EFA | 32.8 (2.8) | 29.3 (3.4) (a) | 31 (0.6) | 31.6 (1.4) (a) | 41.2 (0.6) | 37.9 (2.7) (b) | 33.1 (0.4) | 31.8 (2.1) (a) | **0.0002** |
| MUFA/PUFA | 0.8 (0.1) | 1.1 (0.2) (a) | **0.7 (0.0) (*)** | **0.9 (0.1) (a)** | **0.7 (0.0) (*)** | **0.9 (0.1) (ab)** | 0.7 (0.0) | 0.7 (0.0) (b) | **0.0195** |
| EPA/DHA | 6.3 (0.7) | 7.2 (0.6) (a) | 4.8 (0.6) | 5.7 (0.5) (b) | 11.3 (0.7) | 13.8 (2.6) (c) | 9.2 (0.9) | 6.2 (0.6) (ac) | **0.0004** |
| UI | 225.4 (13.3) | 211.9 (15.0) (a) | 215.6 (2.8) | 218.3 (6.1) (a) | **257.2 (2.0) (*)** | **241.1 (10.1) (b)** | 227.9 (1.9) | 222.9 (7.8) (a) | **0.0014** |
| Σ 20:2 & 22:2 NMI | 5.2 (0.9) | 4.8 (0.7) (a) | **3.2 (0.3) (*)** | **4.2 (0.3) (ac)** | **1.0 (0.3) (*)** | **2.5 (0.7) (b)** | 2.7 (0.4) | 4.2 (0.8) (c) | **0.0002** |
| Σ Unknow PUFA | **2.3 (0.5) (*)** | **1.1 (0.1) (a)** | **3.1 (0.3) (*)** | **2 (0.1) (bc)** | **1.2 (0.1) (*)** | **1.6 (0.3) (a)** | 2.8 (0.2) | 3.9 (0.4) (c) | **0.0001** |

|  | *Astarte* *moerchi* | | *Hiatella* *arctica* | | *Mya* *truncata* | | *Musculus* *discors* | | p-perm |
| --- | --- | --- | --- | --- | --- | --- | --- | --- | --- |
|  | Summer | Winter | Summer | Winter | Summer | Winter | Summer | Winter |  |
| 14:0 | 0.6 (0.2) | 0.5 (0.0) (a) | 2.6 (0.3) | 3.0 (0.3) (b) | 0.8 (0.1) | 0.9 (0.1) (c) | 1.7 (0.1) | 1.8 (0.4) (d) | **0.0001** |
| 15:0 | 0.4 (0.0) | 0.5 (0.0) (a) | 0.9 (0.0) | 0.8 (0.0) (b) | 0.6 (0.1) | 0.8 (0.1) (b) | 0.9 (0.1) | 0.9 (0.2) (b) | **0.0001** |
| 16:0 | 12.8 (1.4) | 13.4 (0.9) (a) | **8.3 (0.7) (*)** | **6.0 (0.4) (b)** | **10 (0.5) (*)** | **14 (0.7) (ac)** | 13.9 (1.1) | 17.6 (2.6) (d) | **0.0001** |
| 17:0 | 1.0 (0.1) | 1.1 (0.1) (a) | 0.3 (0.0) | 0.3 (0.0) (b) | **0.3 (0.0) (*)** | **0.4 (0.0) (bc)** | tr | 0.3 (0.1) (d) | **0.0001** |
| 18:0 | 7.1 (1.3) | 7.3 (0.7) (a) | **10.5 (0.8) (*)** | **8.1 (0.5) (b)** | **2.9 (0.1) (*)** | **3.8 (0.3) (c)** | 7.3 (1.4) | 11.9 (3.7) (a) | **0.0003** |
| 20:0 | 0.4 (0.1) | 0.4 (0.1) (a) | tr | tr (b) | nd | nd (c) | tr | tr (d) | **0.0001** |
| 22:0 | 0.8 (0.2) | 0.6 (0.1) | 0.4 (0.1) | 0.3 (0.1) | 0.4 (0.0) | 0.5 (0.1) | 0.5 (0.1) | 0.6 (0.2) | 0.0652 |
| Σ SFA | 23.2 (2.8) | 24 (1.6) (a) | **23.1 (1.7) (*)** | **18.5 (1.1) (ab)** | **15.1 (0.6) (*)** | **20.4 (1) (b)** | 24.6 (2.5) | 33.2 (6) (c) | **0.0015** |
| 16:1n7 | 2.9 (0.4) | 2.6 (0.2) (a) | 2.5 (0.2) | 2.5 (0.2) (ac) | 2.8 (0.2) | 3.7 (0.4) (a) | 2.0 (0.2) | 2.1 (0.2) (b) | **0.0007** |
| 17:1 | tr | tr (a) | 4.1 (0.6) | 3.5 (0.7) (b) | 1.6 (0.2) | 2.1 (0.5) (c) | 6.3 (0.5) | 4.6 (0.7) (d) | **0.0001** |
| 18:1ω7 | 10.0 (1.5) | 7.5 (1.3) (a) | 1.9 (0.1) | 2.0 (0.1) (be) | **1.7 (0.1) (*)** | **2.3 (0.2) (ce)** | 1.1 (0.2) | 1.3 (0.2) (d) | **0.0001** |
| 18:1ω9 | 2.0 (0.3) | 1.9 (0.1) (a) | 1.1 (0.1) | 0.8 (0.1) (be) | 0.9 (0.1) | 1.2 (0.2) (ce) | 1.2 (0.2) | 1.3 (0.2) (de) | **0.0001** |
| 20:1ω7 | 6.6 (0.4) | 6.6 (0.4) (a) | 6.1 (0.2) | 6.5 (0.3) (a) | 2.3 (0.1) | 2.4 (0.1) (b) | 2.6 (0.2) | 2.8 (0.3) (b) | **0.0001** |
| 20:1ω9 | 1.5 (0.2) | 1.5 (0.2) (a) | 1.5 (0.1) | 1.2 (0.1) (a) | **2.6 (0.1) (*)** | **3.2 (0.1) (b)** | 4.5 (0.1) | 3.8 (0.6) (c) | **0.0001** |
| 20:1ω11 | 10.8 (0.8) | 12 (0.8) (a) | 1.1 (0.0) | 1.2 (0.1) (b) | 7.7 (0.5) | 6.4 (0.4) (c) | 1.0 (0.0) | 1.2 (0.1) (b) | **0.0001** |
| 22:1ω9 | 0.6 (0.1) | 0.4 (0.1) (a) | **0.3 (0.0) (*)** | **0.2 (0.0) (b)** | 0.4 (0.0) | 0.4 (0.1) (a) | 0.4 (0.1) | 0.5 (0.1) (a) | **0.0169** |
| 22:1ω11 | 0.6 (0.1) | 0.5 (0.1) (a) | 0.3 (0.0) | 0.3 (0.1) (b) | 0.2 (0.0) | 0.3 (0.0) (b) | 0.3 (0.1) | 0.3 (0.1) (b) | **0.0001** |
| Σ MUFA | 35 (1.6) | 33.2 (2.1) (a) | 19 (0.9) | 18.3 (0.8) (b) | 20.1 (0.6) | 22 (0.7) (c) | 19.3 (1.1) | 17.7 (1.2) (d) | **0.0001** |
| 18:2ω6 | 1.5 (0.2) | 1.1 (0.1) (a) | **2.0 (0.3) (*)** | **3.8 (0.5) (b)** | **9.5 (0.9) (*)** | **3.8 (1.5) (c)** | 3.0 (0.3) | 3.0 (0.7) (b) | **0.0001** |
| 18:3ω3 | **0.4 (0.1) (*)** | **tr (a)** | **0.2 (0.0) (*)** | **nd (a)** | nd | nd (b) | tr | tr (a) | **0.0001** |
| 20:2 | 0.3 (0.1) | 0.2 (0) (a) | **0.5 (0.0) (*)** | **0.4 (0.0) (b)** | 0.4 (0.0) | 0.5 (0.0) (bc) | 0.4 (0.0) | 0.4 (0.1) (a) | **0.0015** |
| 20:2ω | 7.8 (0.7) | 8.0 (0.6) (a) | **8.4 (0.3) (*)** | **12 (0.8) (b)** | 13 (0.9) | 11.8 (0.7) (c) | 12.3 (0.7) | 11.5 (1.2) (c) | **0.0001** |
| 20:3ω6 | tr | tr (a) | tr | nd (a) | 0.2 (0.0) | 0.4 (0.1) (b) | tr | tr (a) | **0.0001** |
| 20:4ω6 | 4.6 (1.1) | 4.0 (1.1) | 4.6 (0.3) | 5.2 (0.3) | 3.7 (0.4) | 3.4 (0.3) | 4.5 (0.4) | 3.3 (0.4) | 0.3872 |
| 20:5ω3 | 3.9 (0.8) | 3.7 (1.2) (a) | 8.2 (0.5) | 8.4 (0.5) (b) | **10.9 (0.8) (*)** | **14.9 (1.9) (c)** | 11.2 (0.7) | 8.7 (2.2) (bc) | **0.0001** |
| 22:2ω | 6.2 (0.6) | 6.9 (0.4) (a) | **3.8 (0.2) (*)** | **4.7 (0.2) (b)** | **1.0 (0.1) (*)** | **0.7 (0.1) (c)** | 7.6 (0.3) | 6.7 (0.8) (a) | **0.0001** |
| 22:2ωbis | **7.5 (0.5) (*)** | **9.2 (0.5) (a)** | **0.2 (0.0) (*)** | **nd (b)** | tr | tr (c) | nd | nd (a) | **0.0001** |
| 22:6ω3 | 3.0 (0.8) | 3.6 (1.2) (a) | 15.7 (1.2) | 15.2 (0.8) (b) | **16.2 (0.5) (*)** | **12.5 (0.7) (b)** | 9.9 (0.9) | 7.6 (1.4) (c) | **0.0001** |
| Σ PUFA | 39 (3) | 40.1 (3.2) (a) | 55.3 (2.4) | 61 (1.6) (bc) | 63.7 (1.1) | 56.2 (1.5) (b) | **55.2 (2.4) (*)** | **47.1 (5.5) (c)** | **0.0001** |
| 15:0iso | tr | tr (a) | 1.1 (0.1) | 1.0 (0.1) (b) | nd | nd (c) | nd | nd (c) | **0.0001** |
| 16:0iso | 0.8 (0.1) | 1.0 (0.1) (a) | 0.7 (0.1) | 0.6 (0.1) (b) | 0.4 (0.0) | 0.4 (0.1) (c) | **tr (*)** | **0.6 (0.2) (c)** | **0.0001** |
| 17:0anteiso | 0.4 (0) | 0.4 (0) (a) | tr | tr (b) | tr | 0.2 (0.0) (b) | tr | 0.2 (0.1) (b) | **0.0001** |
| 18:0iso | 0.7 (0.1) | 0.7 (0.1) (a) | 0.4 (0.1) | 0.4 (0.0) (b) | 0.4 (0.0) | 0.5 (0.1) (b) | 0.5 (0.1) | 0.6 (0.2) (ac) | **0.0011** |
| Σ BrFA | 2.0 (0.2) | 2.2 (0.2) (a) | 2.4 (0.2) | 2.1 (0.2) (b) | 1.0 (0.1) | 1.1 (0.2) (c) | 0.8 (0.1) | 1.4 (0.4) (c) | **0.0001** |
| TMTD | 0.8 (0.2) | 0.5 (0.1) (a) | 0.2 (0.1) | tr (b) | tr | 0.4 (0.2) (b) | tr | 0.5 (0.2) (ac) | **0.0008** |
| Σ EFA | 11.5 (2.5) | 11.3 (3.4) (a) | 28.6 (1.8) | 28.8 (1.4) (b) | 30.8 (0.6) | 30.8 (1.4) (b) | 25.6 (1.7) | 19.6 (3.5) (c) | **0.0001** |
| MUFA/PUFA | 1.0 (0.1) | 0.9 (0.1) (a) | 0.4 (0.0) | 0.3 (0.0) (b) | **0.3 (0) (*)** | **0.4 (0) (b)** | 0.4 (0.0) | 0.4 (0.0) (b) | **0.0001** |
| EPA/DHA | **1.6 (0.2) (*)** | **1.2 (0.1) (a)** | 0.5 (0.0) | 0.6 (0.0) (b) | **0.7 (0.1) (*)** | **1.2 (0.2) (c)** | 1.2 (0.1) | 1.2 (0.2) (ac) | **0.0001** |
| UI | 139.4 (11.8) | 140.9 (13.5) (a) | 204.5 (9.9) | 213.8 (6.5) (b) | **235.4 (2.5) (*)** | **219.9 (6.1) (c)** | 199.6 (9.5) | 164.0 (22.0) (b) | **0.0001** |
| Σ 20:2 & 22:2 NMI | 22.0 (1.4) | 24.6 (1.3) (a) | **13.0 (0.4) (*)** | **17.1 (0.8) (b)** | 14.7 (0.8) | 13.2 (0.8) (b) | 20.2 (0.9) | 18.7 (1.9) (c) | **0.0001** |
| Σ Unknow PUFA | 3.5 (0.6) | 3.2 (0.5) (a) | 11.2 (0.4) | 11.4 (0.4) (b) | 8.6 (0.3) | 8.1 (0.5) (c) | 6.2 (0.4) | 5.7 (0.4) (d) | **0.0001** |

**Table S3**
